# Protein expression, survival and docetaxel benefit in node-positive breast cancer treated with adjuvant chemotherapy in the FNCLCC - PACS 01 randomized trial

**DOI:** 10.1186/bcr3051

**Published:** 2011-11-01

**Authors:** Jocelyne Jacquemier, Jean-Marie Boher, Henri Roche, Benjamin Esterni, Daniel Serin, Pierre Kerbrat, Fabrice Andre, Pascal Finetti, Emmanuelle Charafe-Jauffret, Anne-Laure Martin, Mario Campone, Patrice Viens, Daniel Birnbaum, Frédérique Penault-Llorca, François Bertucci

**Affiliations:** 1 Department of BioPathology, Institut Paoli-Calmettes, Centre de Recherche en Cancérologie de Marseille, UMR891 Inserm, 232, Bd Ste-Marguerite, Marseille, 13009, France; 2 Department of Molecular Oncology, Institut Paoli-Calmettes, Centre de Recherche en Cancérologie de Marseille, UMR891 Inserm, 232, Bd Ste-Marguerite, Marseille, 13009, France; 3 Department of Biostatistics, Institut Paoli-Calmettes, Centre de Recherche en Cancérologie de Marseille, UMR891 Inserm, 232, Bd Ste-Marguerite, Marseille, 13009, France; 4 Department of Medical Oncology, Institut Claudius Régaud, 20/24, rue du Pont-Saint-Pierre, Toulouse, 31052, France; 5 Department of Medical Oncology, Institut Sainte-Catherine, 1750 Chemin Lavarin, Avignon, 84000, France; 6 Department of Medical Oncology, Centre Eugène Marquis, Rue de la Bataille Flandre-Dunkerque, Rennes, 35042, France; 7 Department of Medical Oncology, Institut Gustave Roussy, 114 rue Edourad Vaillant, Villejuif, 94805, France; 8 UFR of Medicine, Aix-Marseille University, 58 bd Charles Livon, Marseille, 13001, France; 9 Fédération Nationale des Centres de Lutte Contre le Cancer, 101, rue de Tolbiac, Paris, 75654, France; 10 Department of Medical Oncology, Centre René Gauducheau, Bd Jacques Monod, Saint-Herblain, 44805, France; 11 Department of Medical Oncology, Institut Paoli-Calmettes, Centre de Recherche en Cancérologie de Marseille, UMR891 Inserm, 232, Bd Ste-Marguerite, Marseille, 13009, France; 12 Department of Pathology, Centre Jean Perrin, 58, rue Montalembert, Clermont-Ferrand, 63011, France

**Keywords:** adjuvant docetaxel, breast cancer, Ki67, molecular subtypes

## Abstract

**Introduction:**

The PACS01 trial has demonstrated that a docetaxel addition to adjuvant anthracycline-based chemotherapy improves disease-free survival (DFS) and overall survival of node-positive early breast cancer (EBC). We searched for prognostic and predictive markers for docetaxel's benefit.

**Methods:**

Tumor samples from 1,099 recruited women were analyzed for the expression of 34 selected proteins using immunohistochemistry. The prognostic and predictive values of each marker and four molecular subtypes (luminal A, luminal B, HER2-overexpressing, and triple-negative) were tested.

**Results:**

Progesterone receptor-negativity (HR = 0.66; 95% CI 0.47 to 0.92, *P *= 0.013), and Ki67-positivity (HR = 1.53; 95% CI 1.12 to 2.08, *P *= 0.007) were independent adverse prognostic factors. Out of the 34 proteins, only Ki67-positivity was associated with DFS improvement with docetaxel addition (adjusted HR = 0.51, 95% CI 0.33 to 0.79 for Ki67-positive *versus *HR = 1.10, 95% CI 0.75 to 1.61 for Ki67-negative tumors, *P *for interaction = 0.012). Molecular subtyping predicted the docetaxel benefit, but without providing additional information to Ki67 status. The luminal A subtype did not benefit from docetaxel (HR = 1.16, 95% CI 0.73 to 1.84); the reduction in the relapse risk was 53% (HR = 0.47, 95% CI 0.22 to 1.01), 34% (HR = 0.66, 95% CI 0.37 to 1.19), and 12% (HR = 0.88, 95% CI 0.49 to 1.57) in the luminal B, HER2-overexpressing, and triple-negative subtypes, respectively.

**Conclusions:**

In patients with node-positive EBC receiving adjuvant anthracycline-based chemotherapy, the most powerful predictor of docetaxel benefit is Ki67-positivity.

## Introduction

The use of adjuvant chemotherapy has improved prognosis of early breast cancer [1]. Benefits in terms of survival, first demonstrated in the 1970s with the CMF (cyclophosphamide, methotrexate, fluorouracil) regimen, were improved with the addition of anthracycline in the 1980s [2]. Recently, third-generation regimens, based on the addition of taxane, were shown as even more efficient [3].

However, patients do not benefit equally from the same drugs and regimens. Current histo-clinical prognostic factors and the factors predictive for response to a given therapy are not sufficient to solve this heterogeneity, basing the choice of adjuvant chemotherapy regimen upon the risks of relapse and toxicity, comorbidities and physician's experience, rather than upon the probability of efficiency. Today, no factor predictive for efficiency of third-generation regimens has been validated, and all node-positive patients empirically receive adjuvant regimens based on anthracycline and taxane [3], although the optimal role for taxanes in this setting remains controversial. The 20 first-generation taxane trials reported to date that compared taxane-based *versus *taxane-free adjuvant regimens [4-22] and three meta-analyses [23-25] have shown that the taxane-associated absolute benefit is modest, that is, a mere 5% for disease-free survival (DFS) and 3% for overall survival (OS). Taxanes are associated with many side effects and with greater deterioration of quality of life [26]. They are expensive and data are scarce regarding their long-term toxicity. Meta-analyses of first-generation taxane trials have shown that the DFS benefit associated with taxane addition is independent of age and menopausal status, degree of node involvement, estrogen receptor (ER) expression, type of taxane and schedule of administration [24]. In the absence of clear guidance on which patients may benefit from taxanes, their inclusion in adjuvant regimens has also been advocated as a means to reduce exposure to anthracyclines and the risk of associated late toxicity. Furthermore, the type of taxane (docetaxel or paclitaxel), dose and schedule are still debated. Today, an important question is whether subgroups of patients benefit more or less from taxanes in the adjuvant setting. This benefit likely depends upon molecular determinants that remain to be defined.

Randomized clinical trials provide an opportunity for identifying such predictive biomarkers in the adjuvant setting. To date, only immunohistochemistry (IHC)-based studies have been retrospectively reported. Five of them analyzed one to four markers (including ER, progesterone receptor (PR), HER2, and/or Ki67) in a series ranging from 798 to 3,329 tumor samples [9,18,27-31]. A few data suggest that taxanes might benefit ER-negative and/or HER2-positive and/or luminal B breast cancer patients [9,28,29], but results are inconsistent, notably with a recent study [32], negative for 15 proteins analyzed in 1,350 samples.

The PACS01 trial was a multicenter, prospective, randomized, phase III, open-label trial comparing six cycles of fluorouracil, epirubicin (100 mg/m^2^), and cyclophosphamide (FEC) with three cycles of FEC followed by three cycles of docetaxel (100 mg/m^2^; FEC-D), as adjuvant chemotherapy in node-positive operable breast cancer [8]. A total of 1,999 patients were enrolled between 1997 and 2000. With a median follow-up of 60 months, the five-year DFS was 73% with FEC and 78% with FEC-D (18% reduction in the relative risk of relapse). In an analysis restricted to ER-positive tumors, Ki67 expression identified a subgroup of patients who could benefit from docetaxel [30]. Here, we have analyzed the expression of 34 selected IHC markers in a subset of 1,099 patients included in the trial, regardless of their ER status. Our objective was to assess the prognostic and/or predictive value of these markers and the molecular subtypes for the benefit of docetaxel in terms of DFS.

## Materials and methods

### Patients

This biomarker study is ancillary to the PACS01 trial. A tumor block representative of the primary tumor was collected for 1,190 out of the 1,999 enrolled patients. All samples were obtained from operated tumors before any systemic therapy. Patients provided written informed consent for research use, and the study was approved by the ethics committee/institutional review board. Post-menopausal women with hormone receptor (HR)-positive tumors (ER and/or PR-positive) received tamoxifen after completion of chemotherapy. In December 1998, the protocol was amended to require tamoxifen for pre-menopausal women with HR-positive disease. Radiotherapy was mandatory for all patients who had undergone breast-conservative surgery, and was recommended after mastectomy. No patient with an HER2-positive tumor received trastuzumab in the adjuvant setting. The primary end-point was DFS, defined as the time from randomization until the first event: relapse (local, regional, or metastatic), contralateral breast cancer, or death from any cause.

For translational studies, the 1,190 tumors had been centrally immunostained with ER, PR, Ki67 and HER2 specific antibodies on standard slides [30]. Here, we considered ER and PR staining as positive when at least 1% of tumor cells were stained. For Ki67, the positivity cut-off value was 20%. The HER2 status was evaluated with the Dako scale (HercepTest kit scoring guidelines, DakoCytomation, Copenhagen, Denmark): positivity corresponded to 3+ IHC score, or 2+ score with Fluorescent *In Situ *Hybridisation (FISH) amplification. FISH results were obtained from the previously reported centralized reading on standard slides [30]; a HER2/CEP17 ratio higher than 2.2 defined amplification. From the 1,190 cases, a tissue microarray (TMA) was prepared for 1,099 cases. Histo-clinical characteristics and magnitude of docetaxel efficacy in the study group were similar to those of the whole population of the trial (Additional file 1, Table S1), suggesting its representativity. Histo-clinical features of patients whose tumor samples were and were not centrally tested were similar (data not shown).

### Tissue microarrays and immunohistochemistry

Tissue microarrays (TMAs) were prepared as previously described from formalin-fixed and paraffin-embedded tissues [33]. For each tumor, three representative areas were selected from a hematoxylin-eosin-safran stained section of a donor block. Core cylinders with a diameter of 0.6 mm each were punched from each of these areas and deposited into three separate recipient paraffin blocks using a specific arraying device (Alphelys, Plaisir, France). Five-μm sections of the resulting microarray blocks were made and used for IHC after transfer to glass slides.

The selection of the 30 additional proteins to be tested was based on known or putative importance in breast cancer as prognostic/predictive markers or in resistance to taxane, and availability and suitability of a corresponding antibody for paraffin-embedded tissues. They explored different pathways: cell differentiation and adhesion (Cytokeratins CK5/6, CK8/18, CK14, P-Cadherin, E-Cadherin, α-Catenin, β-Catenin, Afadin/AF6, Mucin MUC1, Caveolin CAV1, Moesin, CD10, CD44), proliferation and cell cycle (Aurora A, TACC2, TACC3, Cyclin D1, P21, P27), ER-associated (GATA3), tyrosine kinase signaling (EGFR, FGFR1, MET), apoptosis (BCL2), checkpoints and tumor suppression (P53, PTEN, FHIT), and others (Angiogenin, Topoisomerase II α TOPO2A, microtubule-associated protein TAU).

Immunohistochemical analysis on TMA sections was done as previously described [33] using Dako LSAB^R^2 Kit in the autoimmunostainer (Dako Autostainer, Glostrup, Denmark). Sections were deparaffinized in Histolemon (Carlo Erba Reagenti, Rodano, Italy) and rehydrated in graded ethanol solutions. Details of antibodies are given in Additional file 2 (Table S2). The dilution of each antibody was established on the basis of negative and positive controls and staining with a range of dilutions. For each antibody, the selected titer was in the linear range and allowed the extinction of the negative control and the persistence of the positive control. Results were evaluated by two pathologists (JJ, ECJ) under a light microscope with the Spot Browser system (Alphelys, Plaisir, France). Only invasive tumor components were scored, using the quick score (QS, range from 0 to 300). For each tumor, the mean of the score of a minimum of two core biopsies was calculated. For each antibody, a tumor was considered as positive when the QS was superior to 0.

### Statistical analysis

Distributions of molecular markers and other categorical histo-clinical variables were compared between groups using the Chi*^2 ^*test. The primary end-point was that of the PACS01 trial, DFS, as defined above. Data concerning patients without any event at last follow-up were censored. The follow-up was calculated from the date of randomization to the time of the first event or time of last follow-up for censored patients. Survival curves were derived from Kaplan-Meier estimates and compared by log-rank test. Uni- and multivariate analyses were done using Cox regression analysis. The variables tested in univariate analyses included patients' age, pathological tumor size, number of pathologically involved axillary lymph nodes, Scarff-Bloom Richardson (SBR) grade, and IHC status of the 34 tested markers. The prognostic influence of markers and IHC-defined molecular subtypes was assessed in multivariate analysis by the Cox proportional hazard models, using the adjustment variables preplanned for the PACS01 trial: age, number of pathologically involved axillary lymph nodes, pathological tumor size, SBR grade, and HR status, and the therapeutic arm. The predictive value of each marker and molecular subtype for the docetaxel benefit was assessed using a Cox regression model with terms for treatment by marker interaction, with and without adjustment with the variables quoted above. Survival rates and hazard ratios (HR) are presented with their 95% confidence intervals (95% CI). Statistical tests were two-sided at the 5% level of significance without adjustment for multiple comparisons. All analyses were done using SAS Version 9.1 (Evry-Grégy-sur-Yerres, France). The paper is written in accordance with reporting recommendations for tumor marker prognostic studies (REMARK) criteria.

## Results

### Patients' characteristics and survival

**Figure 1:**
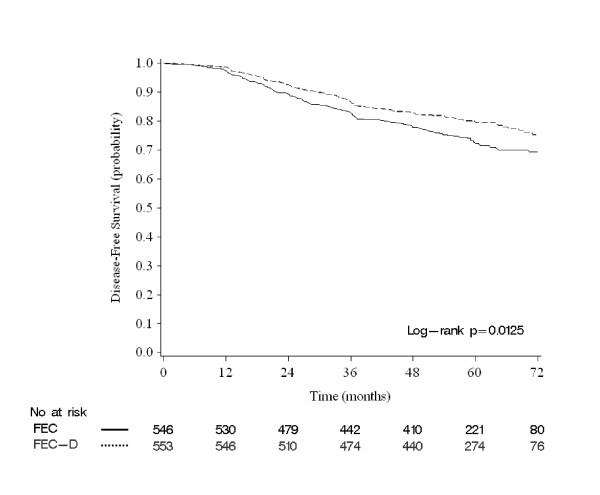
**Disease-free survival in patients included in this sub-study**. Kaplan-Meier DFS curves in patients treated without (FEC: black curve) and with (FEC-D: dashed curve) docetaxel.

Out of the 1,099 cases included in our analysis, 546 had been treated in the FEC arm and 553 in the FEC-D arm. Patients' characteristics were balanced between the two treatment arms, except for the pathological tumor size, more frequently inferior to 20 mm in the FEC-D arm as observed in the PACS01 trial (Table 1). Almost all treated patients received radiotherapy. Tamoxifen use was not different between arms, in both pre- and post-menopausal women. The median follow-up was 60 months. The five-year DFS was 76% (95% CI 73.7 to 78.9). A total of 268 events were reported during the study period, 150 in the FEC arm (27%) and 118 in the FEC-D arm (21%). Respective five-year DFS was 72% (95% CI 68.1 to 76.0) and 79% (95% CI 76.1 to 83.0; *P = *0.0125, log-rank test; Figure 1). In multivariate analysis (Additional file 3, Table S3), the features associated with shorter DFS (Wald test) were age inferior to 50 years (HR = 1.31), more than three involved axillary lymph nodes (HR = 1.94): pathological tumor size superior or equal to 20 mm (HR = 1.78), SBR grade superior to 1 (HR = 1.77 for grade 2, HR = 2.46 for grade 3), and negative hormone receptor status (HR = 1.76), validating the pre-planned choice of these variables as adjustment variables for multivariate analyses. The adjusted HR for an event associated with docetaxel was 0.78 (95% CI 0.60 to 1.02, *P *= 0.066, Wald test), similar to that observed in the whole PACS01 trial.

**Table 1:** Characteristics of patients in this substudy according to the treatment

Characteristic	FEC (*N *= 546)	FEC-D (*N *= 553)	*P*-value
**Age**			0.794
<50 years	261 (47.8%)	260 (47.0%)	
≥50 years	285 (51.2%)	293 (53.0%)	
**Menopausal status**			0.770
Premenopausal	332 (61.8%)	331 (61.0%)	
Postmenopausal	214 (38.2%)	222 (39.0%)	
**Surgery**			
Breast conservation	313 (57.3%)	342 (61.8%)	0.127
Modified mastectomy	233 (42.7%)	211 (38.2%)	
**Pathological tumor size (pT)**			
<2 cm	163 (32.2%)	200 (39.9%)	0.039
2 ≤pT <-5 cm	306 (60.5%)	269 (53.7%)	
≥5 cm	37 (7.3%)	32 (6.4%)	
**SBR Grade**			
I	52 (9.6%)	67 (12.2%)	0.288
II	231 (42.6%)	238 (43.2%)	
III	237 (43.7%)	217 (39.4%)	
Not gradable	22 (4.1%)	29 (5.3%)	
**Positive lymph nodes**	329 (60.3%)	342 (61.8%)	0.589
1 to 3	217 (39.7%)	211 (38.2%)	
≥4			
**Hormone receptors **			
Positive (ER and/or PR)	414 (78.0%)	419 (78.0%)	0.981
Negative (ER and PR)	117 (22.0%)	118 (22.0%)	
**Estrogen receptor **			
Positive	394 (74.2%)	391 (72.8%)	0.607
Negative	137 (25.8%)	146 (27.2%)	
**Progesterone receptor **			
Positive	276 (52.0%)	305 (56.7%)	0.122
Negative	255 (48.0%)	233 (43.3%)	
**HER2 **			
Positive	93 (17.1%)	82 (15.0%)	0.337
Negative	451 (82.9%)	466 (85.0%)	
**DFS, event**			
Yes	150 (27%)	118 (21%)	0.021
No	396 (73%)	435 (79%)	

In most of the subgroups defined according to these histo-clinical prognostic features (age, lymph nodes, size, grade, HR status), addition of docetaxel reduced the risk of relapse, with significantly greater benefit in patients aged 50 years or older, pathological tumor size inferior to 20 mm, four or more positive lymph nodes, and negative HR status (data not shown). However, in multivariate analysis, none of these features showed significant interaction with the chemotherapy arm (Additional file 4, Figure S1).

### Protein markers, survival and docetaxel benefit

The expression of 34 proteins in tumor samples was centrally analyzed by IHC: ER, PR, Ki67 and HER2 on standard slides, and the 30 additional proteins on TMAs. Results are detailed in Table 2. Staining was heterogeneous between tumors. The percent of positive tumors varied from 12.5% for Moesin to 98% for CK8/18.

**Table 2:** Univariate and multivariate analyses of 34 antibodies for DFS

Marker	Category	N (%)	Univariate		Multivariate	
			Unadjusted Hazard Ratio (95%CI)	*P*-value (Log-rank)	Adjusted Hazard Ratio (95%CI)	*P-*value(Wald)
**AF6**	Neg.	193 (23%)				
	Pos.	656 (77%)	0.86 (0.63 to 1.20)	0.386	0.87 (0.61 to 1.24)	0.437
**Angiogenin**	Neg.	71 (7%)				
	Pos.	879 (93%)	1.06 (0.65 to 1.74)	0.803	1.01 (0.59 to 1.75)	0.967
**Aurora A**	Neg.	585 68%)				
	Pos.	276 (32%)	1.41 (1.07 to 1.87)	0.015	1.32 (0.98 to 1.79)	0.070
**BCL2**	Neg.	377 (39%)				
	Pos.	580 (61%)	0.60 (0.47 to 0.78)	<.001	0.82 (0.60 to 1.12)	0.217
**α-Catenin**	Neg.	359 42%)				
	Pos.	501 (58%)	0.88 (0.67 to 1.16)	0.374	0.99 (0.73 to 1.34)	0.927
**β-Catenin**	Neg.	265 (29%)				
	Pos.	636 (71%)	0.83 (0.63 to 1.11)	0.216	0.88 (0.64 to 1.21)	0.439
**CAV1**	Neg.	179 (19%)				
	Pos.	778 (81%)	1.39 (0.96 to 1.99)	0.077	1.16 (0.76 to 1.76)	0.495
**CD10**	Neg.	409 (45%)				
	Pos.	508 (55%)	1.19 (0.91 to 1.55)	0.199	1.20 (0.90 to 1.61)	0.211
**CD44**	Neg.	430 (61%)				
	Pos.	280 (39%)	0.83 (0.62 to 1.14)	0.264	0.90 (0.65 to 1.25)	0.524
**CK5/6**	Neg.	248 (27%)				
	Pos.	667 (73%)	0.88 (0.67 to 1.18)	0.414	0.98 (0.71 to 1.36)	0.921
**CK8/18**	Neg.	22 (2%)				
	Pos.	948 (98%)	0.30 (0.17 to 0.55)	<.001	0.54 (0.28 to 1.03)	0.060
**CK14**	Neg.	774 (83%)				
	Pos.	158 (17%)	1.01 (0.71 to 1.42)	0.974	0.81 (0.55 to 1.19)	0.288
**Cyclin D1**	Neg.	316 (33%)				
	Pos.	648 (67%)	0.86 (0.66 to 1.13)	0.301	1.11 (0.81 to 1.51)	0.526
**E-Cadherin**	Neg.	128 (13%)				
	Pos.	873 (87%)	1.09 (0.74-1.60)	0.653	0.98 (0.62-1.57)	0.937
**EGFR**	Neg.	814 (81%)				
	Pos.	185 (19%)	1.02 (0.74 to 1.41)	0.895	0.72 (0.49 to 1.05)	0.090
**ER**	Neg.	283 (26%)				
	Pos.	785 (74%)	0.53 (0.42 to 0.69)	<0.001	0.90 (0.47 to 1.71)	0.744
**FGFR1**	Neg.	114 (15%)				
	Pos.	626 (85%)	1.36 (0.86 to 2.14)	0.189	1.60 (0.93 to 2.74)	0.087
**FHIT**	Neg.	237 (26%)				
	Pos.	672(74%)	1.03 (0.76 to 1.40)	0.825	1.41 (0.98 to 2.01)	0.062
**GATA3**	Neg.	166 (17%)				
	Pos.	816 (83%)	0.70 (0.52 to 0.96)	0.027	0.83 (0.58 to 1.19)	0.311
**HER2**	Neg.	917 (84%)				
	Pos.	175 (16%)	1.64 (1.23 to 2.19)	<0.001	1.12 (0.80 to 1.57)	0.493
**Ki67**	Neg.	661 (70%)				
	Pos.	280 (30%)	1.97 (1.51 to 2.56)	<0.001	1.53 (1.12 to 2.08)	0.007
**MET**	Neg.	602 (66%)				
	Pos.	315 (34%)	1.26 (0.97 to 1.65)	0.088	1.25 (0.93 to 1.66)	0.134
**Moesin**	Neg.	824 (87%)				
	Pos.	118 (13%)	1.31 (0.91 to 1.89)	0.145	1.95 (.63 to 1.44)	0.815
**MUC1**	Neg.	95 (9%)				
	Pos.	938 (91%)	0.59 (0.42 to 0.86)	0.005	0.77 (0.51 to 1.16)	0.213
**P21**	Neg.	378 (41%)				
	Pos.	545 (59%)	0.93 (0.71 to 1.22)	0.616	0.84 (0.62 to 1.13)	0.241
**P27**	Neg.	175 (19%)				
	Pos.	768 (81%)	0.89 (0.65 to 1.24)	0.509	1.06 (0.75 to 1.50)	0.754
**P53**	Neg.	746 (75%)				
	Pos.	246 (25%)	1.59 (1.21 to 2.07)	<.001	1.29 (0.95 to 1.75)	0.107
**P-Cadherin**	Neg.	570 (61%)				
	Pos.	371 (39%)	1.44 (1.12 to 1.87)	0.005	1.31 (0.96 to 1.78)	0.089
**PR**	Neg.	488 (46%)				
	Pos.	581 (54%)	0.54 (0.42 to 0.69)	<0.001	0.66 (0.47 to 0.92)	0.013
**PTEN**	Neg.	314 (34%)				
	Pos.	610 (66%)	1.00 (0.76 to 1.33)	0.977	1.00 (0.74 to 1.36)	0.991
**TACC2**	Neg.	148 (17%)				
	Pos.	723 (83%)	1.05 (0.73 to 1.52)	0.777	0.95 (0.64 to 1.42)	0.796
**TACC3**	Neg.	35 (6%)				
	Pos.	564 (94%)	1.83 (0.75 to 4.47)	0.179	1.17 (0.47 to 2.88)	0.738
**TAU**	Neg.	685 (83%)				
	Pos.	141 (17%)	0.56 (0.36 to 0.88)	0.011	0.75 (0.47 to 1.21)	0.243
**TOPO2A**	Neg.	199 (22%)				
	Pos.	714 (78%)	1.41 (0.99 to 2.00)	0.052	1.39 (0.93 to 2.07)	0.104

Table 2 reports the correlation between each single marker and DFS. The 12 proteins associated with shorter DFS in univariate analysis (negativity of BCL2, CK8/18, ER, GATA3, MUC1, PR, and TAU, and positivity of Aurora A, HER2, Ki67, P-Cadherin, and P53) were included in multivariate analysis, together with pre-planned histo-clinical variables. The status of two proteins remained associated with shorter DFS (Wald test): PR-negativity (HR = 0.66; 95% CI 0.47 to 0.92, *P *= 0.013), and Ki67-positivity (HR = 1.53; 95% CI 1.12 to 2.08, *P *= 0.007).

**Figure 2:**
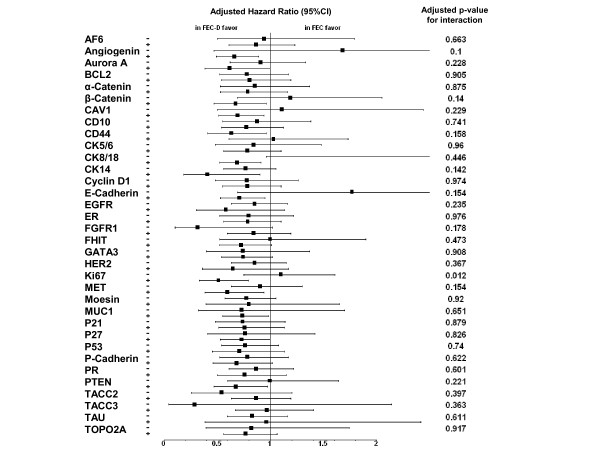
**Adjusted hazard ratios associated with docetaxel addition**. Forest plots showing the adjusted hazard ratios associated with docetaxel addition according to expression of 34 proteins.

**Figure 3:**
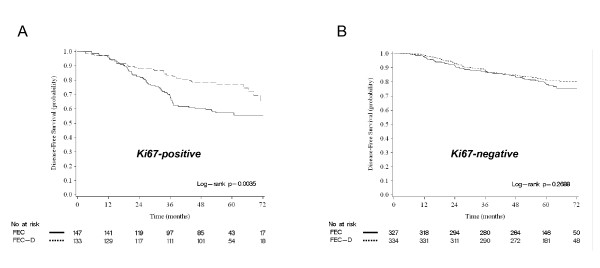
**Disease-free survival according to Ki67 status and docetaxel**. **A**. Kaplan-Meier DFS curves in patients with Ki67-positive status according to docetaxel addition. **B**. Similar to B, but in patients with Ki67-negative status.

We then searched for an association between each protein and the benefit of docetaxel in terms of DFS. Results of univariate and multivariate analyses are detailed in Additional file 5, Table S4. On multivariate analysis (Table 3 and Figure 2), the addition of docetaxel reduced the risk of an event (HR for relapse inferior to 1) in most of the subgroups defined by the positive status and the negative status of most of the 34 proteins, significantly (*P *<0.05) for 11 proteins (Angiogenin, β-Catenin, CAV1, CD44, E-Cadherin, CK8/18, Ki67, MET, MUC1, P27, and PTEN). However, an interaction between the protein expression and the addition of docetaxel was significant only for Ki67 (*P *= 0.012). Docetaxel was associated with a 49% reduction in the risk of an event (HR = 0.51, 95% CI 0.33 to 0.79; *P *= 0.003 Wald test) in Ki67-positive patients (Figure 3A), but with no reduction (HR = 1.10 95% CI 0.75 to 1.61; *P *= 0.612) in Ki67-negative patients (Figure 3B). Interaction of borderline significance (*P *≤0.15) was observed for CK14, Angiogenin, and β-Catenin, with a trend towards docetaxel benefit for patients with a marker-positive tumor.

**Table 3:** Multivariate analyses of 34 antibodies for interaction with chemotherapy arm

Marker	Category	N Arms A/B	Adjusted Hazard ratio (95% CI)	*P*-value forinteraction
AF6	Neg.	99/94	0.94 (0.50 to 1.79)	0.663
	Pos.	321/335	0.86 (0.61 to 1.23)	
Angiogenin	Neg.	34/37	1.68 (0.47 to 5.96)	0.1
	Pos.	439/440	0.66 (0.49 to 0.89)	
Aurora A	Neg.	293/292	0.91 (0.62 to 1.33)	0.228
	Pos.	135/141	0.61 (0.38 to 1.00)	
BCL2	Neg.	184/193	0.78 (0.52 to 1.17)	0.905
	Pos.	285/295	0.80 (0.54 to 1.19)	
α-Catenin	Neg.	177/182	0.85 (0.53 to 1.37)	0.875
	Pos.	250/251	0.79 (0.53 to 1.16)	
β-Catenin	Neg.	123/142	1.19 (0.69 to 2.05)	0.14
	Pos.	324/312	0.67 (0.47 to 0.96)	
CAV1	Neg.	82/97	1.11 (0.50 to 2.44)	0.229
	Pos.	389/389	0.69 (0.51 to 0.94)	
CD10	Neg.	197/212	0.87 (0.55 to 1.38)	0.741
	Pos.	259/249	0.77 (0.54 to 1.12)	
CD44	Neg.	210/220	0.63 (0.41 to 0.96)	0.158
	Pos.	128/152	1.03 (0.61 to 1.73)	
CK5/6	Neg.	122/126	0.84 (0.48 to 1.48)	0.96
	Pos.	332/335	0.79 (0.56 to 1.10)	
CK8/18	Neg.	13/9	4.73 (0.96 to 23.3)	0.446
	Pos.	468/480	0.69 (0.52 to 0.91)	
CK14	Neg.	386/388	0.77 (0.56 to 1.05)	0.142
	Pos.	84/74	0.41 (0.18 to 0.90)	
Cyclin D1	Neg.	161/155	0.78 (0.48 to 1.26)	0.974
	Pos.	317/331	0.78 (0.55 to 1.10)	
E-Cadherin	Neg.	62/66	1.77 (0.69 to 4.59)	0.154
	Pos.	432/441	0.71 (0.53 to 0.95)	
EGFR	Neg.	401/413	0.85 (0.63 to 1.16)	0.235
	Pos.	95/90	0.58 (0.30 to 1.13)	
ER	Neg.	137/146	0.79 (0.52 to 1.22)	0.976
	Pos.	394/391	0.79 (0.56 to 1.10)	
FGFR1	Neg.	56/58	0.32 (0.10 to 1.02)	0.178
	Pos.	306/320	0.84 (0.59 to 1.19)	
FHIT	Neg.	127/110	0.00 (0.52 to 1.90)	0.473
	Pos.	321/351	0.73 (0.52 to 1.01)	
GATA3	Neg.	88/78	0.74 (0.40 to 1.37)	0.908
	Pos.	407/409	0.74 (0.54 to 1.02)	
HER2	Neg.	451/466	0.85 (0.63 to 1.15)	0.367
	Pos.	93/82	0.65 (0.36 to 1.17)	
Ki67	Neg.	327/334	1.10 (0.75 to 1.61)	0.012
	Pos.	147/133	0.51 (0.33 to 0.79)	
MET	Neg.	301/301	0.91 (0.63 to 1.30)	0.154
	Pos.	150/165	0.59 (0.38 to 0.94)	
Moesin	Neg.	412/412	0.77 (0.57 to 1.05)	0.92
	Pos.	56/62	0.80 (0.39 to 1.65)	
MUC1	Neg.	53/42	0.73 (0.32 to 1.70)	0.651
	Pos.	461/477	0.73 (0.55 to 0.98)	
P21	Neg.	187/191	0.74 (0.48 to 1.14)	0.879
	Pos.	272/273	0.76 (0.51 to 1.13)	
P27	Neg.	78/97	0.76 (0.41 to 1.42)	0.826
	Pos.	378/390	0.73 (0.53 to 1.00)	
P53	Neg.	364/382	0.76 (0.54 to 1.08)	0.74
	Pos.	123/123	0.71 (0.45 to 1.13)	
P-Cadherin	Neg.	282/288	0.78 (0.52 to 1.17)	0.622
	Pos.	179/192	0.68 (0.46 to 1.02)	
PR	Neg.	255/233	0.86 (0.61 to 1.22)	0.601
	Pos.	276/305	0.76 (0.50 to 1.15)	
PTEN	Neg.	152/162	0.99 (0.60 to 1.64)	0.221
	Pos.	315/295	0.68 (0.47 to 0.97)	
TACC2	Neg.	70/78	0.54 (0.25 to 1.20)	0.397
	Pos.	366/357	0.87 (0.63 to 1.19)	
TACC3	Neg.	13/22	0.29 (0.04 to 2.14)	0.363
	Pos.	284/280	0.97 (0.67 to 1.41)	
TAU	Neg.	340/345	0.83 (0.59 to 1.16)	0.611
	Pos.	65/76	0.96 (0.38 to 2.42)	
TOPO2A	Neg.	104/95	0.82 (0.39 to 1.74)	0.917
	Pos.	359/355	0.77 (0.56 to 1.06)	

### Molecular subtypes, survival and docetaxel benefit

Genomics has revealed at least four subtypes of breast cancer: luminal A, luminal B, basal, and HER2/ERBB2-overexpressing [34]. These subtypes may be approximately, but more easily for clinical routine, defined by four IHC markers (ER, PR, HER2, and Ki67). Based on these markers, we classified the 1,099 samples into four subtypes: luminal A (HR-positive, HER2-negative, Ki67-negative: *N *= 525, 54%), luminal B (HR-positive, HER2-negative, Ki67-positive; *N *= 125, 13%), HER2-overexpressing (HER2-positive, whatever HR; *N *= 175, 18%), and triple-negative (HR-negative, HER2-negative; *N *= 148, 15%).

As expected, these subtypes correlated with histo-clinical variables (Additional file 6, Table S5). Triple-negative tumors and HER2-overexpressing tumors were more frequently grade 3 than luminal tumors (*P *<0.0001), as were luminal B tumors when compared with luminal A tumors. HER2-overexpressing tumors presented more frequently more than three involved axillary lymph nodes, followed by luminal B tumors, then luminal A, then triple-negative tumors (*P *= 0.015). Classical basal markers (CK14, EGFR, Moesin, P-Cadherin, P53), were more frequently positive in triple-negative tumors (*P *≤0.001). Five-year DFS was different among the subtypes (*P *<0.0001, log-rank test): 83% for luminal A (95% CI 79.4to 6.2), 73% for luminal B (95% CI 63.8 to 79.8), 66% for HER2-overexpressing (95% CI 58.3 to 72.7), and 65% for triple-negative (95% CI 56.9 to 72.4).

The benefit of docetaxel was analyzed per subtype (Additional file 7, Figure S2). Results of uni- and multivariate analyses are shown in Additional file 8, Table S6. In multivariate analysis, docetaxel was associated with a 53% reduction in the risk of relapse in the luminal B subtype (HR = 0.47, 95% CI 0.22 to 1.01; *P *= 0.05, Wald test), a 34% reduction in the HER2-overexpressing subtype (HR = 0.66, 95% CI 0.37 to 1.19; *P *= 0.14), and a 12% reduction (HR = 0.88, 95% CI 0.49 to 1.57, *P *= 0.67) in the triple-negative subtype. By contrast, the risk of an event was 16% higher with *vs *without docetaxel in luminal A tumors (HR = 1.16, 95% CI 0.73 to 1.84, *P *= 0.52). The interaction between benefit of docetaxel and each subtype was significant for luminal B (*P *= 0.047), borderline for HER2-overexpressing (*P *= 0.14), and not significant for triple-negative (*P *= 0.46).

We explored the added predictive value of molecular subtyping compared with Ki67 status alone by testing the interaction between the molecular subtype factor and the treatment arm in a Cox model adjusted for the pre-planned histo-clinical variables and Ki67 by treatment interaction. Analysis using the likelihood ratio test did not show any significant added predictive value to that provided by the Ki67 status alone for DFS (*P *= 0.88).

## Discussion

Identifying the patients who would and those who would not benefit from taxanes is crucial to moving away from the "one shoe fits all" strategy. Here, we have analyzed the expression of 34 selected proteins in a subset of 1,099 patients included in the PACS01 trial.

We show that a Ki67-positive status is not only independently associated with shorter DFS, but also with the benefit of a docetaxel addition in women treated with adjuvant anthracycline-based chemotherapy. Ki67, expressed during the cell cycle, is a well-established cell proliferation marker. Its expression in breast cancer correlates with poor prognosis [35,36] and higher response to chemotherapy. In the neo-adjuvant setting, correlation between Ki67 positivity and response to taxanes, either as monotherapy [37] or in association with anthracyclines [38], has been reported, although the relationship was not observed in other small series [39,40]. We found a prognostic correlation much more important in docetaxel-free patients (*P *<0.001, log-rank test) than in docetaxel-treated patients (*P *= 0.048), in relation to the interaction observed with docetaxel benefit. Such interaction has been previously studied in randomized trials of adjuvant chemotherapy. Higher efficiency of the CMF regimen *vs*. no chemotherapy (*P *= 0.16 for interaction) was reported in Ki67-positive ER-positive patients treated in the NSABP-20 trial [41], whereas Ki67 labeling index was not predictive of better response to adjuvant chemotherapy in endocrine-responsive tumors [42]. Bartlett *et al*. analyzed data of the UK NEAT/BR9601 trial, which showed benefit for the addition of anthracyclines to CMF regimen, and did not detect any interaction with anthracycline benefit for Ki67 status [43]. The benefit of docetaxel (*P *= 0.11 for interaction) in Ki67-positive ER-positive patients enrolled in the PACS01 trial has been reported [30]. Our present analysis, applied to all available PACS01 samples regardless of the ER status, showed a significant interaction, with an adjusted HR for the risk of relapse in Ki67-positive patients (HR = 0.51) equal to that previously reported for ER-positive patients only, and superior to that observed in the unselected whole population (HR = 0.81). By contrast, in an analysis of the BCIRG001 trial [32], Ki67 did not present any interaction with docetaxel benefit. In this study, the positivity threshold was the median value of the tested population, whereas we used the more classical 20% cut-off [36]. We did not find in the literature any pre-clinical or clinical data in the neo-adjuvant or the metastatic setting regarding Ki67 status and the response to or the benefit associated with docetaxel specifically. Of course, our observations will require validation before application in routine. But already, they clearly suggest that patients with Ki67-positive breast cancer potentially derive a high benefit from adjuvant docetaxel (adjusted HR = 0.51), and it might be a candidate for intensifying taxane delivery (six cycles and/or a dose-dense scheme). By contrast, we did not observe any additional benefit for docetaxel in patients with Ki67-negative tumors (adjusted HR = 1.10), who represent more than two-thirds of the tested patients. A potential application might be to omit docetaxel for these patients and go back to six cycles of FEC. However, because that would increase the risk of cardiotoxicity and leukemia, it is reasonable to think that the delivery of three FEC three docetaxel cycles would remain "as good" as six FEC cycles and worthwhile giving. Whether Ki67 is mechanistically involved in the response to docetaxel remains to be demonstrated, but even if it is not, it may be a marker of a phenotype more sensitive to docetaxel. One may suppose that docetaxel, which exerts its cytotoxic effects in the G2/M phase of the cycle by inhibiting the microtubule disassembly (antimitotic effect), is more active on rapidly proliferating cells (high Ki67).

We did not find any interaction among the 33 other markers and docetaxel benefit. In fact, the relapse risk was decreased by docetaxel addition in nearly all subgroups defined according to these markers, but without significant difference for benefit between the marker-positive and marker-negative subgroups. Univariate analysis showed higher reduction of risk in ER-negative patients than in ER-positive ones, but the interaction was not significant. Similar observation was reported in the pooled analysis of two randomized trials [27], and the analyses of BCIRG001 [32], NSABP-B28 [44], CALGB9344 [45], and TACT [9] trials. Today, the predictive value of ER status for response to taxanes is not demonstrated when considering ER alone, and additional markers are required for identifying subgroups of ER-positive and ER-negative who most benefit from these drugs. Our previous [30] and present data suggest that Ki67 is a potential candidate. This predictive value is also debated for HER2. We found a significant interaction with HER2 status in univariate analysis, with more benefit of docetaxel addition in HER2-positive patients (HR for relapse: 0.46 *vs *0.84), but the interaction lost significance in multivariate analysis. Analysis of two other adjuvant trials reported a benefit from the addition of taxanes in HER2-positive patients [9,28], with significant interaction in one [28], but analyses of other trials yielded conflicting results [16,18,32,46]. Regarding the other markers, we observed a trend towards a benefit from docetaxel in patients whose tumor was positive for CK14, Angiogenin, and β-Catenin, with a statistically borderline interaction, which deserves further analysis in larger series.

More complex molecular combinations could prove more informative than single markers for predicting docetaxel benefit [9,29-31,47]. Here, we have defined molecular subtypes according to the status of ER, PR, HER2 and Ki67 proteins. These subtypes displayed expected histo-clinical features. Notably, five-year DFS was relatively good in the luminal A subtype, poor in HER2-overexpressing and the triple-negative subtypes, and intermediate in the luminal B subtype, in close agreement with survival rates observed in the BCIRG001 [29] and GEICAM9906 trials [31], even if the definition of luminal B and HER2-overexpressing subtypes was a little different. Clearly, the subtype that did not benefit from docetaxel in our series was luminal A, as reported in the BCIRG001 trial [29], and for ER-positive, HER2-negative tumors (assimilated to luminal A) in the CALGB9344 [28] and TACT [9] trials. By contrast, luminal A tumors unexpectedly benefited from paclitaxel in the GEICAM9906 trial [18]. The reasons for discrepancy between this latter observation and the former ones are unclear, and longer follow-up is required. The benefit we observed in the triple-negative tumors (adjusted HR = 0.88) was not different from the benefit observed in the whole-population, and interaction was not significant. Two subtypes benefited from docetaxel addition: luminal B and HER2-overexpressing. In luminal B patients, the absolute benefit in five-year DFS was 19% (DFS: 64% with FEC *vs *83% with FEC-D); docetaxel reduced the risk of relapse by 53% after adjustment for histo-clinical variables, and the interaction was significant. Similarly, luminal B BCIRG001 patients showed a 44% reduction of relapse risk with docetaxel [29]. This was not confirmed in the GEICAM9906 sub-study [31], which, however, used the same definition as the BCIRG001 trial (HR-positive, and either Ki67-positive or HER2-positive). In these two studies, the positivity cut-off was similar for ER and PR (1%), but different for Ki67 (10% in the GEICAM9906, 13% in the BCIRG001 study). From literature data and our comparison of luminal A vs luminal B tumors (Additional file 6, Table S5), some biological features of luminal B vs luminal A tumors might speculatively explain this differential benefit from docetaxel: not only the higher proliferation rate (Ki67), but also the lesser expression of BCL2 and TAU whose expression has been associated with taxane resistance [48,49], and the higher expression of P53, whose mutations have been associated with resistance to DNA-damaging agents such as anthracyclines [50], and relative sensitivity to taxanes [51]. However, none of these markers, except Ki67, was associated with docetaxel benefit in our univariate analysis (Additional file 5, Table S4). Finally, docetaxel led to a 34% adjusted reduction of the relapse risk (HR = 0.66) in the HER2-overexpressing subtype, with a borderline interaction (*P *= 0.14). No adjuvant trastuzumab was given in our series, but it is likely that trastuzumab would not decrease the observed docetaxel benefit. Significant interaction between HER2, treatment and outcome was found in the CALGB9344 trial, independently of ER status [28]. Higher benefit of taxanes was also reported in HER2-positive, HR-negative BCIRG001 [29] and TACT [9] patients, but not in the GEICAM9906 trial [31]. Because the current IHC definition of luminal B and HER2-overexpressing subtypes is not consensual, we repeated analyses using definitions used by others in the BCIRG001 [29] and GEICAM9906 trials [31]: luminal B tumors were defined as HR-positive/HER2-negative/Ki67-positive or HR-positive/HER2-positive (*N *= 206, 21%), and HER2-overexpressing tumors as HR-negative/HER2-positive (*N *= 86, 9%). Luminal A and triple-negative subtypes were not changed. As shown in Additional file 9, Table S7, unadjusted and adjusted HR for relapse were very similar to previous analysis regarding the benefit of docetaxel per subtype, with more benefit in the luminal B and HER2-overexpressing subtypes, and no benefit in the luminal A subtype..

Our study presents several strengths (randomized prospective trial, high number of samples representative of the whole trial population and of tested proteins, including novel markers), but, like retrospective subset biomarker studies reported in adjuvant trials, suffers also from several limitations: a relatively small number of analyzed markers when compared with high-throughput profiling of frozen samples, a relatively limited proportion of available samples (55%), and limitations intrinsic to unplanned analyses. The relatively low number of events and the relatively small benefit of the experimental arm (HR = 0.81) make the study not powered enough to detect small interactions between markers and docetaxel benefit. The PACS01 trial, like other trials, was not designed to detect the benefit of taxanes in patient subgroups defined by markers. Conversely, unplanned analyses confer a risk of false positive results. For both reasons, meta-analysis of first-generation taxane trials incorporating molecular data, ideally centrally generated, is warranted in the context of international collaborations (planned future EBCTCG meta-analysis), as well as validation in ongoing second-generation taxane trials. For example, a validation study is planned to test the predictive value of Ki67 in the PACS04 trial, which compared three cycles of FEC three of docetaxel *vs*. six of ED (Epirubicine Docetaxel).

Other limitations are methodological (IHC) and conceptual (breast cancer heterogeneity). Breast cancer is heterogeneous. Given the extent of differences between the four molecular subtypes (luminal A and B, HER2-overexpressing, and triple-negative), and because a signal relevant in a given subtype may be diluted and undetectable in the whole population, and conversely a signal relevant in the whole population may not be detected in a given subtype, another promising approach is to redefine prognostic and predictive markers in each subtype [52]. Results of univariate prognostic analyses are detailed in Additional file 10, Table S8, showing, for example, that the negativity of BCL2, EGFR, and TAU were significant prognosticators in the luminal A subtype, but not in the other subtypes, or that TACC2-positivity had an unfavorable prognostic value in the HER2-overexpressing subtype, but favorable in the triple-negative subtype. However, given the relatively small size of each subtype in our series, this deserves to be reassessed in a larger series and in multivariate analysis.

Regarding IHC, it is known to be non-quantitative, and it is difficult to know the most biologically relevant cut-offs for prognostic and predictive analyses. In the present study, all cut-offs were predefined before statistical analyses. For ER, PR and HER2, we used the Saint-Gallen cut-offs [53]. For the other proteins, the challenge is more substantial. For Ki67, important guidelines are under development [42,54-57]. A meta-analysis of 46 studies published in 2007 reported many different Ki67 cut-offs, ranging from 3.5% to 34% [35]. The 2011 Saint-Gallen cut-off has been fixed at 14%, based on comparison with gene array data (PAM50) as a prognostic factor [54]. However, as stated in the recommendations [53], the "optimal cut-points in Ki67 labeling index for prediction of efficacy of endocrine or cytotoxic therapy may vary". Here, to remain consistent with our previous PACS01 sub-study [30], we used a 20% cut-off. For the other 30 tested proteins, and in the absence of consensual guidelines, we used a 1% cut-off. We are aware that the chosen cut-offs may not be optimal in terms of prognostic and/or predictive values. We have thus planned the analysis of different cut-offs for each protein, which will require identification and validation in independent series. In the present series, the prognostic value of Ki67 and its value predictive for docetaxel benefit remained significant in multivariate analysis when we applied a 15% cut-off (data not shown), regardless of the cut-off for ER and PR: 1% (*P *= 0.028 and *P *for interaction = 0.033 respectively) or 10% (*P *= 0.022 and *P *for interaction = 0.031 respectively). By contrast, the favorable impact of the luminal B subtype for the docetaxel benefit lost its significance (*P*-value for interaction = 0.208 in multivariate analysis) when we used a 15% cut-off for Ki67 (Additional file 11, Table S9). Another analytic approach is to study the prognostic and predictive values of continuous IHC variables. As a preliminary approach, we used quartiles of IHC measurements to categorize our population (percent of stained tumor cells for the data generated on standard slides and quick score QS for the TMA data; Additional file 12, Table S10). We then assessed for each protein the magnitude of the prognostic and treatment effects as a function of such categorization. The results of multivariate analyses (Additional file 13, Table S11) showed a differential DFS according to the value of Ki67 and PR, with a maximum difference for the highest quartile (effect unfavorable for Ki67 and favorable for PR). There was also a trend towards a differential benefit of docetaxel versus no docetaxel according to the value of Ki67 and Aurora A, with a maximum benefit for the highest quartile for both proliferation markers (Additional file 14, Table S12). Complementary analyses, such as STEPP analysis (subpopulation treatment effect pattern plot) [58], are warranted.

## Conclusions

We confirm that ER, PR or HER2 status alone does not predict the benefit of docetaxel as adjuvant therapy in node-positive early breast cancer patients treated with anthracycline-based chemotherapy. We show that Ki67 status alone may be informative. We show also that the molecular subclassification may predict which patient may not benefit from docetaxel (luminal A) and may benefit more than the unselected population (luminal B, HER2-overexpressing), but does not provide additional information to Ki67 status alone. To go further in analyses, a supervised analysis [33] will be applied to our dataset, using semi-quantitative and quantitative data, to attempt identifying a multiprotein predictor for docetaxel benefit, overall and per subtype.

## Abbreviations

CMF: cyclophosphamide: methotrexate: fluorouracil; DFS: disease-free survival; EBC: early breast cancer; ER: estrogen receptor; FEC: fluorouracil: epirubicin: cyclophosphamide; FEC-D: FEC-docetaxel; FISH: Fluorescent *In Situ *Hybridisation; HR: hormone receptor; IHC: immunohistochemistry; OS: overall survival; PR: progesterone receptor; QS: quick score; SBR: Scarff-Bloom Richardson; STEPP: subpopulation treatment effect pattern plot; TMA: tissue microarray.

## Competing interests

The authors declare that they have no competing interests.

## Authors' contributions

JJ and FB designed the concept of the study. HR, DS, PK, FA, MC, PV and FB contributed to the accrual of patients in the trial. JJ, ECJ and FPL participated in the central pathological reading of samples, and carried out IHC analyses. JMB, BE and PF performed the statistical analyses. ALM collected histo-clinical data and samples. JJ, DB and FB were responsible for interpretation of results. JJ and FB wrote the manuscript. All authors read and approved the final manuscript.

## Supplementary Material

Additional file 1 **Table S1 (WORD file)**. Characteristics of patients in the PACS01 trial and in this sub-study. Click here for file

Additional file 2 **Table S2 (WORD file)**. Antibodies used for immunohistochemistry. Click here for file

Additional file 3 **Table S3 (WORD file)**. Multivariate analysis of DFS according to histo-clinical variables. Click here for file

Additional file 4 **Figure S1 (POWER POINT file)**. Adjusted hazard ratios associated with docetaxel addition (forest plots) according to histo-clinical variables. Click here for file

Additional file 5 **Table S4 (WORD file)**. Univariate and multivariate analyses of 34 antibodies for interaction with chemotherapy arm. Click here for file

Additional file 6 **Table S5 (WORD file)**. Molecular subtypes and correlations with histo-clinical features and IHC results. Click here for file

Additional file 7 **Figure S2 (POWER POINT file)**. Disease-free survival according to molecular subtypes and docetaxel. Kaplan-Meier DFS curves in patients with luminal A (A), luminal B (B), HER2-overexpressing (C) and triple-negative (D) tumor according to docetaxel addition. Click here for file

Additional file 8 **Table S6 (WORD file)**. Univariate and multivariate analyses of molecular subtypes for interaction with chemotherapy arm. Click here for file

Additional file 9 **Table S7 (WORD file)**. Univariate and multivariate analyses of molecular subtypes for interaction with chemotherapy arm, using another definition for luminal B and HER2-overexpressing subtypes. Click here for file

Additional file 10 **Table S8 (WORD file)**. Univariate analyses of 34 antibodies for DFS per subtype. Click here for file

Additional file 11 **Table S9 (WORD file)**. Univariate and multivariate analyses of molecular subtypes for interaction with chemotherapy arm, using 15% cut-off for Ki67 for the definition of luminal subtypes. Click here for file

Additional file 12 **Table S10 (WORD file)**. Range and quartiles used for each antibody. Click here for file

Additional file 13 **Table S11 (WORD file)**. Univariate and multivariate analyses "per quartiles" for DFS Click here for file

Additional file 14 **Table S12 (WORD file)**. Univariate and multivariate analyses "per quartiles" for interaction with chemotherapy arm. Click here for file

## References

[B1] Early Breast Cancer Trialists' Collaborative Group Effects of chemotherapy and hormonal therapy for early breast cancer on recurrence and 15-year survival: an overview of the randomized trials Lancet 2005 365 1687 1717 15894097 10.1016/S0140-6736(05)66544-0

[B2] Sparano JA Hortobagyi GN Gralow JR Perez EA Comis RL Recommendations for research priorities in breast cancer by the Coalition of Cancer Cooperative Groups Scientific Leadership Council: systemic therapy and therapeutic individualization Breast Cancer Res Treat 2010 119 511 527 10.1007/s10549-009-0433-y 19526354

[B3] Bedard PL Di Leo A Piccart-Gebhart MJ Taxanes: optimizing adjuvant chemotherapy for early-stage breast cancer Nat Rev Clin Oncol 2010 7 22 36 10.1038/nrclinonc.2009.186 19997076

[B4] Buzdar AU Singletary SE Valero V Booser DJ Ibrahim NK Rahman Z Theriault RL Walters R Rivera E Smith TL Holmes FA Hoy E Frye DK Manuel N Kau SW McNeese MD Strom E Thomas E Hunt K Ames F Berry D Hortobagyi GN Evaluation of paclitaxel in adjuvant chemotherapy for patients with operable breast cancer: preliminary data of a prospective randomized trial Clin Cancer Res 2002 8 1073 1079 12006521

[B5] Henderson IC Berry DA Demetri GD Cirrincione CT Goldstein LJ Martino S Ingle JN Cooper MR Hayes DF Tkaczuk KH Fleming G Holland JF Duggan DB Carpenter JT Frei E Schilsky RL Wood WC Muss HB Norton L Improved outcomes from adding sequential Paclitaxel but not from escalating Doxorubicin dose in an adjuvant chemotherapy regimen for patients with node-positive primary breast cancer J Clin Oncol 2003 21 976 983 10.1200/JCO.2003.02.063 12637460

[B6] Mamounas EP Bryant J Lembersky B Fehrenbacher L Sedlacek SM Fisher B Wickerham DL Yothers G Soran A Wolmark N Paclitaxel after doxorubicin plus cyclophosphamide as adjuvant chemotherapy for node-positive breast cancer: results from NSABP B-28 J Clin Oncol 2005 23 3686 3696 10.1200/JCO.2005.10.517 15897552

[B7] Martin M Pienkowski T Mackey J Pawlicki M Guastalla JP Weaver C Tomiak E Al-Tweigeri T Chap L Juhos E Guevin R Howell A Fornander T Hainsworth J Coleman R Vinholes J Modiano M Pinter T Tang SC Colwell B Prady C Provencher L Walde D Rodriguez-Lescure A Hugh J Loret C Rupin M Blitz S Jacobs P Murawsky M Adjuvant docetaxel for node-positive breast cancer N Engl J Med 2005 352 2302 2313 10.1056/NEJMoa043681 15930421

[B8] Roche H Fumoleau P Spielmann M Canon JL Delozier T Serin D Symann M Kerbrat P Soulié P Eichler F Viens P Monnier A Vindevoghel A Campone M Goudier MJ Bonneterre J Ferrero JM Martin AL Genève J Asselain B Sequential adjuvant Epirubicin-based and Docetaxel Chemotherapy for node-positive breast cancer patients: the PACS01 trial J Clin Oncol 2006 24 5664 5671 10.1200/JCO.2006.07.3916 17116941

[B9] Ellis P Barrett-Lee P Johnson L Cameron D Wardley A O'Reilly S Verrill M Smith I Yarnold J Coleman R Earl H Canney P Twelves C Poole C Bloomfield D Hopwood P Johnston S Dowsett M Bartlett JM Ellis I Peckitt C Hall E Bliss JM Sequential docetaxel as adjuvant chemotherapy for early breast cancer (TACT): an open-label, phase III, randomised controlled trial Lancet 2009 373 1681 1692 10.1016/S0140-6736(09)60740-6 19447249 PMC2687939

[B10] Joensuu H Kellokumpu-Lehtinen PL Bono P Alanko T Kataja V Asola R Utriainen T Kokko R Hemminki A Tarkkanen M Turpeenniemi-Hujanen T Jyrkkio S Flander M Helle L Ingalsuo S Johansson K Jaaskelainen AS Pajunen M Rauhala M Kaleva-Kerola J Salminen T Leinonen M Elomaa I Isola J Adjuvant docetaxel or vinorelbine with or without trastuzumab for breast cancer N Engl J Med 2006 354 809 820 10.1056/NEJMoa053028 16495393

[B11] Burnell M Levine MN Chapman JA Bramwell V Gelmon K Walley B Vandenberg T Chalchal H Albain KS Perez EA Rugo H Pritchard K O'Brien P Shepherd LE Cyclophosphamide, epirubicin, and Fluorouracil versus dose-dense epirubicin and cyclophosphamide followed by Paclitaxel versus Doxorubicin and cyclophosphamide followed by Paclitaxel in node-positive or high-risk node-negative breast cancer J Clin Oncol 2009 28 77 82 19901117 10.1200/JCO.2009.22.1077 PMC2799234

[B12] Evans TR Yellowlees A Foster E Earl H Cameron DA Hutcheon AW Coleman RE Perren T Gallagher CJ Quigley M Crown J Jones AL Highley M Leonard RC Mansi JL Phase III randomized trial of doxorubicin and docetaxel versus doxorubicin and cyclophosphamide as primary medical therapy in women with breast cancer: an anglo-celtic cooperative oncology group study J Clin Oncol 2005 23 2988 2995 10.1200/JCO.2005.06.156 15860854

[B13] Fountzilas G Skarlos D Dafni U Gogas H Briasoulis E Pectasides D Papadimitriou C Markopoulos C Polychronis A Kalofonos HP Siafaka V Kosmidis P Timotheadou E Tsavdaridis D Bafaloukos D Papakostas P Razis E Makrantonakis P Aravantinos G Christodoulou C Dimopoulos AM Postoperative dose-dense sequential chemotherapy with epirubicin, followed by CMF with or without paclitaxel, in patients with high-risk operable breast cancer: a randomized phase III study conducted by the Hellenic Cooperative Oncology Group Ann Oncol 2005 16 1762 1771 10.1093/annonc/mdi366 16148021

[B14] Francis P Crown J Di Leo A Buyse M Balil A Andersson M Nordenskjold B Lang I Jakesz R Vorobiof D Gutierrez J van Hazel G Dolci S Jamin S Bendahmane B Gelber RD Goldhirsch A Castiglione-Gertsch M Piccart-Gebhart M Adjuvant chemotherapy with sequential or concurrent anthracycline and docetaxel: Breast International Group 02-98 randomized trial J Natl Cancer Inst 2008 100 121 133 10.1093/jnci/djm287 18182617

[B15] Gianni L Baselga J Eiermann W Porta VG Semiglazov V Lluch A Zambetti M Sabadell D Raab G Cussac AL Bozhok A Martinez-Agullo A Greco M Byakhov M Lopez JJ Mansutti M Valagussa P Bonadonna G Phase III trial evaluating the addition of paclitaxel to doxorubicin followed by cyclophosphamide, methotrexate, and fluorouracil, as adjuvant or primary systemic therapy: European Cooperative Trial in Operable Breast Cancer J Clin Oncol 2009 27 2474 2481 10.1200/JCO.2008.19.2567 19332727

[B16] Jones S Holmes FA O'Shaughnessy J Blum JL Vukelja SJ McIntyre KJ Pippen JE Bordelon JH Kirby RL Sandbach J Hyman WJ Richards DA Mennel RG Boehm KA Meyer WG Asmar L Mackey D Riedel S Muss H Savin MA Docetaxel With cyclophosphamide is associated with an overall survival benefit compared with doxorubicin and cyclophosphamide: 7-year follow-up of US Oncology Research Trial 9735 J Clin Oncol 2009 27 1177 1183 10.1200/JCO.2008.18.4028 19204201

[B17] Goldstein LJ O'Neill A Sparano JA Perez EA Shulman LN Martino S Davidson NE Concurrent doxorubicin plus docetaxel is not more effective than concurrent doxorubicin plus cyclophosphamide in operable breast cancer with 0 to 3 positive axillary nodes: North American Breast Cancer Intergroup Trial E 2197 J Clin Oncol 2008 26 4092 4099 10.1200/JCO.2008.16.7841 18678836 PMC2654376

[B18] Martin M Rodriguez-Lescure A Ruiz A Alba E Calvo L Ruiz-Borrego M Munarriz B Rodriguez CA Crespo C de Alava E Lopez Garcia-Asenjo JA Guitian MD Almenar S Gonzalez-Palacios JF Vera F Palacios J Ramos M Gracia Marco JM Lluch A Alvarez I Segui MA Mayordomo JI Anton A Baena JM Plazaola A Modolell A Pelegri A Mel JR Aranda E Adrover E Randomized phase 3 trial of fluorouracil, epirubicin, and cyclophosphamide alone or followed by Paclitaxel for early breast cancer J Natl Cancer Inst 2008 100 805 814 10.1093/jnci/djn151 18505968

[B19] Polyzos A Malamos N Boukovinas I Adamou A Ziras N Kalbakis K Kakolyris S Syrigos K Papakotoulas P Kouroussis C Karvounis N Vamvakas L Christophyllakis C Athanasiadis A Varthalitis I Georgoulias V Mavroudis D FEC versus sequential docetaxel followed by epirubicin/cyclophosphamide as adjuvant chemotherapy in women with axillary node-positive early breast cancer: a randomized study of the Hellenic Oncology Research Group (HORG) Breast Cancer Res Treat 2010 119 95 104 10.1007/s10549-009-0468-0 19636702

[B20] Cognetti F De Laurentiis M De Matteis A Manzione L Boni C Palazzo S Di Palma M Papaldo P De Placido S Bianco AR Sequential epirubicin-docetaxel-CMF as adjuvant therapy for node-positive early breast cancer: updated results of the TAXIt216 randomized trial Ann Oncol 2008 19 a1820

[B21] Del Mastro L Costantini M Durando A Michelotti A Danese S Aitini E Olmeo N Pronzato P Venturini M Intergruppo GONO-M Cyclophosphamide, epirubicin, and 5-fluorouracil versus epirubicin plus paclitaxel in node-positive early breast cancer patients: A randomized, phase III study of Gruppo Oncologico Nord Ovest-Mammella Intergruppo Group J Clin Oncol 2008 26 a516

[B22] Nitz U Huober JB Lisboa B Harbeck N Fischer H Moebus V Hoffmann G Augustin D Weiss E Kuhn W West German Study Group/AGO-Mamma Interim results of Intergroup EC-Doc Trial: a randomized multicenter phase III trial comparing adjuvant CEF/CMF to EC- docetaxel in patients with 1-3 positive lymph nodes [abstract] J Clin Oncol 2008 26 a515 10.1200/JCO.2007.13.8131

[B23] Bria E Nistico C Cuppone F Carlini P Ciccarese M Milella M Natoli G Terzoli E Cognetti F Giannarelli D Benefit of taxanes as adjuvant chemotherapy for early breast cancer: pooled analysis of 15,500 patients Cancer 2006 106 2337 2344 10.1002/cncr.21886 16649217

[B24] De Laurentiis M Cancello G D'Agostino D Giuliano M Giordano A Montagna E Lauria R Forestieri V Esposito A Silvestro L Pennacchio R Criscitiello C Montanino A Limite G Bianco AR De Placido S Taxane-based combinations as adjuvant chemotherapy of early breast cancer: a meta-analysis of randomized trials J Clin Oncol 2008 26 44 53 10.1200/JCO.2007.11.3787 18165639

[B25] Ferguson T Wilcken N Vagg R Ghersi D Nowak AK Taxanes for adjuvant treatment of early breast cancer Cochrane Database Syst Rev 2007 CD004421 17943815 10.1002/14651858.CD004421.pub2

[B26] Martin M Lluch A Segui MA Ruiz A Ramos M Adrover E Rodriguez-Lescure A Grosse R Calvo L Fernandez-Chacon C Roset M Anton A Isla D del Prado PM Iglesias L Zaluski J Arcusa A Lopez-Vega JM Munoz M Mel JR Toxicity and health-related quality of life in breast cancer patients receiving adjuvant docetaxel, doxorubicin, cyclophosphamide (TAC) or 5-fluorouracil, doxorubicin and cyclophosphamide (FAC): impact of adding primary prophylactic granulocyte-colony stimulating factor to the TAC regimen Ann Oncol 2006 17 1205 1212 10.1093/annonc/mdl135 16766587

[B27] Andre F Broglio K Roche H Martin M Mackey JR Penault-Llorca F Hortobagyi GN Pusztai L Estrogen receptor expression and efficacy of docetaxel-containing adjuvant chemotherapy in patients with node-positive breast cancer: results from a pooled analysis J Clin Oncol 2008 26 2636 2643 10.1200/JCO.2007.14.9146 18509176

[B28] Hayes DF Thor AD Dressler LG Weaver D Edgerton S Cowan D Broadwater G Goldstein LJ Martino S Ingle JN Henderson IC Norton L Winer EP Hudis CA Ellis MJ Berry DA HER2 and response to paclitaxel in node-positive breast cancer N Engl J Med 2007 357 1496 1506 10.1056/NEJMoa071167 17928597

[B29] Hugh J Hanson J Cheang MC Nielsen TO Perou CM Dumontet C Reed J Krajewska M Treilleux I Rupin M Magherini E Mackey J Martin M Vogel C Breast cancer subtypes and response to docetaxel in node-positive breast cancer: use of an immunohistochemical definition in the BCIRG 001 trial J Clin Oncol 2009 27 1168 1176 10.1200/JCO.2008.18.1024 19204205 PMC2667821

[B30] Penault-Llorca F Andre F Sagan C Lacroix-Triki M Denoux Y Verriele V Jacquemier J Baranzelli MC Bibeau F Antoine M Lagarde N Martin AL Asselain B Roche H Ki67 expression and docetaxel efficacy in patients with estrogen receptor-positive breast cancer J Clin Oncol 2009 27 2809 2815 10.1200/JCO.2008.18.2808 19380452

[B31] Martin M Rodriguez-Lescure A Ruiz A Alba E Calvo L Ruiz-Borrego M Santaballa A Rodriguez CA Crespo C Abad M Dominguez S Florian J Llorca C Mendez M Godes M Cubedo R Murias A Batista N Garcia MJ Caballero R de Alava E Molecular predictors of efficacy of adjuvant weekly paclitaxel in early breast cancer Breast Cancer Res Treat 2010 123 149 157 10.1007/s10549-009-0663-z 20037779

[B32] Dumontet C Krajewska M Treilleux I Mackey JR Martin M Rupin M Lafanechere L Reed JC BCIRG 001 molecular analysis: prognostic factors in node-positive breast cancer patients receiving adjuvant chemotherapy Clin Cancer Res 2010 16 3988 3997 10.1158/1078-0432.CCR-10-0079 20576719

[B33] Jacquemier J Ginestier C Rougemont J Bardou VJ Charafe-Jauffret E Geneix J Adelaide J Koki A Houvenaeghel G Hassoun J Maraninchi D Viens P Birnbaum D Bertucci F Protein expression profiling identifies subclasses of breast cancer and predicts prognosis Cancer Res 2005 65 767 779 15705873

[B34] Sorlie T Perou CM Tibshirani R Aas T Geisler S Johnsen H Hastie T Eisen MB van de Rijn M Jeffrey SS Thorsen T Quist H Matese JC Brown PO Botstein D Eystein Lonning P Borresen-Dale AL Gene expression patterns of breast carcinomas distinguish tumor subclasses with clinical implications Proc Natl Acad Sci USA 2001 98 10869 10874 10.1073/pnas.191367098 11553815 PMC58566

[B35] de Azambuja E Cardoso F de Castro G Jr Colozza M Mano MS Durbecq V Sotiriou C Larsimont D Piccart-Gebhart MJ Paesmans M Ki-67 as prognostic marker in early breast cancer: a meta-analysis of published studies involving 12,155 patients Br J Cancer 2007 96 1504 1513 10.1038/sj.bjc.6603756 17453008 PMC2359936

[B36] Stuart-Harris R Caldas C Pinder SE Pharoah P Proliferation markers and survival in early breast cancer: a systematic review and meta-analysis of 85 studies in 32,825 patients Breast 2008 17 323 334 10.1016/j.breast.2008.02.002 18455396

[B37] Chang JC Makris A Gutierrez MC Hilsenbeck SG Hackett JR Jeong J Liu ML Baker J Clark-Langone K Baehner FL Sexton K Mohsin S Gray T Alvarez L Chamness GC Osborne CK Shak S Gene expression patterns in formalin-fixed, paraffin-embedded core biopsies predict docetaxel chemosensitivity in breast cancer patients Breast Cancer Res Treat 2008 108 233 240 10.1007/s10549-007-9590-z 17468949

[B38] Darb-Esfahani S Loibl S Muller BM Roller M Denkert C Komor M Schluns K Blohmer JU Budczies J Gerber B Noske A du Bois A Weichert W Jackisch C Dietel M Richter K Kaufmann M von Minckwitz G Identification of biology-based breast cancer types with distinct predictive and prognostic features: role of steroid hormone and HER2 receptor expression in patients treated with neoadjuvant anthracycline/taxane-based chemotherapy Breast Cancer Res 2009 11 R69 10.1186/bcr2363 19758440 PMC2790846

[B39] Estevez LG Cuevas JM Anton A Florian J Lopez-Vega JM Velasco A Lobo F Herrero A Fortes J Weekly docetaxel as neoadjuvant chemotherapy for stage II and III breast cancer: efficacy and correlation with biological markers in a phase II, multicenter study Clin Cancer Res 2003 9 686 692 12576436

[B40] Sjostrom J Blomqvist C Heikkila P Boguslawski KV Raisanen-Sokolowski A Bengtsson NO Mjaaland I Malmstrom P Ostenstadt B Bergh J Wist E Valvere V Saksela E Predictive value of p53, mdm-2, p21, and mib-1 for chemotherapy response in advanced breast cancer Clin Cancer Res 2000 6 3103 3110 10955790

[B41] Paik S Tang G Shak S Kim C Baker J Kim W Cronin M Baehner FL Watson D Bryant J Costantino JP Geyer CE Jr Wickerham DL Wolmark N Gene expression and benefit of chemotherapy in women with node-negative, estrogen receptor-positive breast cancer J Clin Oncol 2006 24 3726 3734 10.1200/JCO.2005.04.7985 16720680

[B42] Viale G Regan MM Mastropasqua MG Maffini F Maiorano E Colleoni M Price KN Golouh R Perin T Brown RW Kovacs A Pillay K Ohlschlegel C Gusterson BA Castiglione-Gertsch M Gelber RD Goldhirsch A Coates AS Predictive value of tumor Ki-67 expression in two randomized trials of adjuvant chemoendocrine therapy for node-negative breast cancer J Natl Cancer Inst 2008 100 207 212 10.1093/jnci/djm289 18230798

[B43] Bartlett JM Munro AF Dunn JA McConkey C Jordan S Twelves CJ Cameron DA Thomas J Campbell FM Rea DW Provenzano E Caldas C Pharoah P Hiller L Earl H Poole CJ Predictive markers of anthracycline benefit: a prospectively planned analysis of the UK National Epirubicin Adjuvant Trial (NEAT/BR9601) Lancet Oncol 2010 11 266 274 10.1016/S1470-2045(10)70006-1 20079691

[B44] Mamounas EP Bryant J Lembersky B Fehrenbacher L Sedlacek SM Fisher B Wickerham DL Yothers G Soran A Wolmark N Paclitaxel after doxorubicin plus cyclophosphamide as adjuvant chemotherapy for node-positive breast cancer: results from NSABP B-28 J Clin Oncol 2005 23 3686 3696 10.1200/JCO.2005.10.517 15897552

[B45] Berry DA Cirrincione C Henderson IC Citron ML Budman DR Goldstein LJ Martino S Perez EA Muss HB Norton L Hudis C Winer EP Estrogen-receptor status and outcomes of modern chemotherapy for patients with node-positive breast cancer JAMA 2006 295 1658 1667 10.1001/jama.295.14.1658 16609087 PMC1459540

[B46] Kostopoulos I Arapantoni-Dadioti P Gogas H Papadopoulos S Malamou-Mitsi V Scopa CD Markaki S Karagianni E Kyriakou V Margariti A Kyrkou E Pavlakis K Zaramboukas T Skordalaki A Bourli A Markopoulos C Pectasides D Dimopoulos MA Skarlos D Fountzilas G Evaluation of the prognostic value of HER-2 and VEGF in breast cancer patients participating in a randomized study with dose-dense sequential adjuvant chemotherapy Breast Cancer Res Treat 2006 96 251 261 10.1007/s10549-005-9062-2 16538542

[B47] Hayes DF Is there a standard type and duration of adjuvant chemotherapy for early stage breast cancer? Breast 2009 18 S131 134 19914531 10.1016/S0960-9776(09)70287-5

[B48] Andre F Hatzis C Anderson K Sotiriou C Mazouni C Mejia J Wang B Hortobagyi GN Symmans WF Pusztai L Microtubule-associated protein-tau is a bifunctional predictor of endocrine sensitivity and chemotherapy resistance in estrogen receptor-positive breast cancer Clin Cancer Res 2007 13 2061 2067 10.1158/1078-0432.CCR-06-2078 17404087

[B49] Noguchi S Predictive factors for response to docetaxel in human breast cancers Cancer Sci 2006 97 813 820 10.1111/j.1349-7006.2006.00265.x 16805818 PMC11158941

[B50] Aas T Borresen AL Geisler S Smith-Sorensen B Johnsen H Varhaug JE Akslen LA Lonning PE Specific P53 mutations are associated with de novo resistance to doxorubicin in breast cancer patients Nat Med 1996 2 811 814 10.1038/nm0796-811 8673929

[B51] Wahl AF Donaldson KL Fairchild C Lee FY Foster SA Demers GW Galloway DA Loss of normal p53 function confers sensitization to Taxol by increasing G2/M arrest and apoptosis Nat Med 1996 2 72 79 10.1038/nm0196-72 8564846

[B52] Pusztai L Gene expression profiling of breast cancer Breast Cancer Res 2009 11 S11 20030862 10.1186/bcr2430 PMC2797691

[B53] Goldhirsch A Wood WC Coates AS Gelber RD Thurlimann B Senn HJ Strategies for subtypes--dealing with the diversity of breast cancer: highlights of the St Gallen International Expert Consensus on the Primary Therapy of Early Breast Cancer 2011 Ann Oncol 2011 22 1736 1747 10.1093/annonc/mdr304 21709140 PMC3144634

[B54] Cheang MC Chia SK Voduc D Gao D Leung S Snider J Watson M Davies S Bernard PS Parker JS Perou CM Ellis MJ Nielsen TO Ki67 index, HER2 status, and prognosis of patients with luminal B breast cancer J Natl Cancer Inst 2009 101 736 750 10.1093/jnci/djp082 19436038 PMC2684553

[B55] Urruticoechea A Smith IE Dowsett M Proliferation marker Ki-67 in early breast cancer J Clin Oncol 2005 23 7212 7220 10.1200/JCO.2005.07.501 16192605

[B56] Viale G Giobbie-Hurder A Regan MM Coates AS Mastropasqua MG Dell'Orto P Maiorano E MacGrogan G Braye SG Ohlschlegel C Neven P Orosz Z Olszewski WP Knox F Thurlimann B Price KN Castiglione-Gertsch M Gelber RD Gusterson BA Goldhirsch A Prognostic and predictive value of centrally reviewed Ki-67 labeling index in postmenopausal women with endocrine-responsive breast cancer: results from Breast International Group Trial 1-98 comparing adjuvant tamoxifen with letrozole J Clin Oncol 2008 26 5569 5575 10.1200/JCO.2008.17.0829 18981464 PMC2651094

[B57] Yerushalmi R Woods R Ravdin PM Hayes MM Gelmon KA Ki67 in breast cancer: prognostic and predictive potential Lancet Oncol 2010 11 174 183 10.1016/S1470-2045(09)70262-1 20152769

[B58] Bonetti M Gelber RD A graphical method to assess treatment-covariate interactions using the Cox model on subsets of the data Stat Med 2000 19 2595 2609 10.1002/1097-0258(20001015)19:19<2595::AID-SIM562>3.0.CO;2-M 10986536

