# Supplementary material for: Protein expression, survival and docetaxel benefit in node-positive breast cancer treated with adjuvant chemotherapy in the FNCLCC - PACS 01 randomized trial
Source: Breast Cancer Res. 2011 Nov 1;13(6):R109. doi: 10.1186/bcr3051 (PMC3326551; doi:10.1186/bcr3051)
Supplement: Additional file 1 — Table S1 (WORD file). Characteristics of patients in the PACS01 trial and in this sub-study. [file bcr3051-S1.DOC]

**Suppl. Table 1: Characteristics of patients in the PACS01 trial and in this substudy.**

| **Characteristic** | **Substudy**  **(N = 1099)** | **PACS01 trial**  **(N = 1999)** |
| --- | --- | --- |
|  | N° of patients (%) | |
| **Age**  < 50 years  ≥ 50 years | 521 (47.4%)  578 (52.6%) | 1005 (50.3%)  994 (49.7%) |
| **Menopausal status**  Premenopausal  Postmenopausal | 663 (61.4%)  436 (38.6%) | 1211 (62.4%)  788 (37.6%) |
| **Surgery** |  |  |
| Breast conservation | 655 (59.6%) | 510 (51.2%) |
| Modified mastectomy | 444 (40.4%) | 486 (48.8%) |
| **Pathological tumour size (pT)** |  |  |
| < 2 cm | 363 (36.0%) | 673 (36.8%) |
| 2 ≤ pT <-5 cm | 575 (57.1%) | 1014 (55.5%) |
| ≥ 5 cm | 69 (6.9%) | 141 (7.7%) |
| **SBR Grade** |  |  |
| I | 119 (10.9%) | 228 (11.6%) |
| II | 469 (42.9%) | 868 (44.3%) |
| III | 454 (41.5%) | 774 (39.5%) |
| Not gradable | 51 (4.7%) | 91 (4.6%) |
| **Positive lymph nodes** |  |  |
| 1 - 3 | 671 (61.1%) | 1237 (61.9%) |
| ≥ 4 | 428 (38.9%) | 762 (38.1%) |
| **Hormone receptor** |  |  |
| Positive (ER and/or PR) | 833 (78.0%) | 1562 (79.0%) |
| Negative (ER and PR) | 235 (22.0%) | 414 (30.0%) |
| **Estrogen receptor** |  |  |
| Positive | 785 (73.5%) | 1472 (74.0%) |
| Negative | 283 (26.5%) | 518 (26.0%) |
| **Progesterone receptor*** |  |  |
| Positive | 581 (54.4%) | 1283 (64.9%) |
| Negative | 488 (45.6%) | 695 (35.1%) |
| **HER2** |  |  |
| Positive | 175 (16.0%) | -- |
| Negative | 917 (84.0%) | -- |
| **DFS, event** |  |  |
| Yes  No | 268 (24.3%)  831 (75.7%) | 482 (24.1%)  1517 (75.9%) |

*: difference significant between centralised reading (substudy) and local reading in each centre (PACS01 trial)
