# Supplementary material for: Protein expression, survival and docetaxel benefit in node-positive breast cancer treated with adjuvant chemotherapy in the FNCLCC - PACS 01 randomized trial
Source: Breast Cancer Res. 2011 Nov 1;13(6):R109. doi: 10.1186/bcr3051 (PMC3326551; doi:10.1186/bcr3051)
Supplement: Additional file 2 — Table S2 (WORD file). Antibodies used for immunohistochemistry. [file bcr3051-S2.DOC]

**Suppl. Table 2: Antibodies used for immunohistochemistry.**

| **Protein (acronym)** | **Antibody** | **Origin** | **Clone** | **Pretreatment** | **Dilution** | **Threshold** |
| --- | --- | --- | --- | --- | --- | --- |
| Afadin (AF6) | Mmab | Transduction laboratories | 35 | DTRS  (40 min, 98°C) | 1/50° | Any positive cell |
| Angiogenin | Mmab | Santa Cruz | SC9044 | Citrate buffer  (40min, 98°C) | 1/20° | Any positive cell |
| Aurora A (STK6/STK15) | Mmab | C. Prigent,  Rennes | / | DTRS  (40 min, 98°C) | 1/25° | Any positive cell |
| BCL2 | Mmab | Dako  Corporation | 124 | Citrate buffer  (40min, 98°C) | 1/100° | Any positive cell |
| α -Catenin (CTNNA1) | Mmab | Zymed  Laboratories | α CAT-7A4 | Citrate buffer  (40min, 98°C) | 1/200° | Any positive cell |
| β -Catenin (CTNNB1) | Mmab | Transduction laboratories | 14 | Citrate buffer  (40min, 98°C) | 1/2500° | Any positive cell |
| Caveolin (CAV1) | Ppab | Santa Cruz Biotechnology | N20 | Citrate buffer  (40min, 98°C | 1/100° | Any positive cell |
| CD10 | Mmab | Novocastra Laboratories | 56C6 | EDTA | 1/100° | Any positive cell |
| CD44 | Mmab | Neomarkers | 156-c11 | Citrate buffer  (40min, 98°C | 1/100° | Any positive cell |
| Cyclin D1 (CCND1) | Mmab | Zymed  laboratories | AM29 | Citrate buffer  (40min, 98°C) | 1/200° | Any positive cell |
| Cytokeratins 5 and 6 (CK5/6) | Mmab | Dako  Corporation | D5/16B4 | DTRS  (40 min, 98°C) | 1/10° | Any positive cell |
| Cytokeratins 8 and 18 (CK8/18) | Mmab | Zymed  Laboratories | Zym5.2 | DTRS  (40 min, 98°C) | 1/200° | Any positive cell |
| Cytokeratin 14 (CK14) | Mmab | Novocastra | L1002 | Citrate buffer  (40min, 98°C) | 1/30° | Any positive cell |
| E-Cadherin (CDH1) | Mmab | Transduction Laboratories | 36 | Citrate buffer  (40min, 98°C) | 1/2000° | Any positive cell |
| Epidermal growth factor receptor (EGFR) | Mmab | Zymed  Laboratories | 31G7 | Pepsin  (30 min, 37°C) | 1/20° | Any positive cell |
| Estrogen receptor (ER)* | Mmab | Novocastra Laboratories | 6F11 | Citrate buffer  (40min, 98°C) | 1/60° | Any positive cell |
| Fibroblast growth factor receptor 1 (FGFR1) | Rpab | Santa Cruz Biotechnology | Sc-121 | DTRS  (40 min, 98°C) | 1/200° | Any positive cell |
| Fragile histidine triad (FHIT) | Rpab | Zymed  Laboratories | ZR44 | Citrate buffer  (40min, 98°C) | 1/300° | Any positive cell |
| GATA3 | Mmab | Santa Cruz Biotechnology | Sc-268 | Citrate buffer  (40min, 98°C) | 1/100° | Any positive cell |
| HER2* | Mmab | Novocastra Laboratories | CB 11 | Citrate buffer  (40min, 98°C) | 1/500° | 2+ (FISH+) or 3+ |
| Ki67* | Mmab | Dako  Corporation | Ki-67 | Citrate buffer  (40min, 98°C) | 1/100° | Positive cells >20% |
| MET | Mmab | Zymed | 3D4 | Citrate buffer  (40min, 98°C | 1/50° | Any positive cell |
| Moesin | Mmab | Biomeda | 38/87 | Citrate buffer  (40min, 98°C) | 1/400° | Any positive cell |
| Mucin 1 (MUC1) | Mmab | Transgene | H23 | None | 1/1000° | Any positive cell |
| P21 | Mmab | Oncogene research | EA 10 | Citrate buffer  (40min, 98°C) | 1/20° | Any positive cell |
| P27 | Mmab | Dako | SX53G8 | Citrate buffer  (40min, 98°C) | 1/100° | Any positive cell |
| P53 | Mmab | Immunotech | DO-1 | Citrate buffer  (40min, 98°C) | 1/20° | Any positive cell |
| P-Cadherin (CDH3) | Mmab | Transduction Laboratories | 56 | DTRS  (40 min, 98°C) | 1/75 | Any positive cell |
| Progesterone receptor (PR)* | Mmab | Dako  Corporation | PgR 636 | Citrate buffer  (40min, 98°C) | 1/80 | Any positive cell |
| PTEN | Ppab | Cell signaling | 26H9 | Citrate buffer  (40min, 98°C) | 1/1000° | Any positive cell |
| Transforming acidic coiled-coil 2/ Taxin 2 (TACC2) | Rpab | Upstate Biotechnology | 07-228 | DTRS  (40 min, 98°C) | 1/40 | Any positive cell |
| Transforming acidic coiled-coil 3/ Taxin 3 (TACC3) | Rpab | Upstate Biotechnology | 07-233 | DTRS  (40 min, 98°C) | 1/100 | Any positive cell |
| TAU | Rpab | Dako  Corporation | A0024 | None | 1/500° | Any positive cell |
| Topoisomerase II α | Mmab | Novocastra | 3F6 | Citrate buffer  (40min, 98°C | 1/20° | Any positive cell |

Note: Mmab: mouse monoclonal antibody; Rpab: rabbit polyclonal antibody; DTRS: Dako target retrieval solution.

*, analysis using standard slides.
