# Supplementary material for: Protein expression, survival and docetaxel benefit in node-positive breast cancer treated with adjuvant chemotherapy in the FNCLCC - PACS 01 randomized trial
Source: Breast Cancer Res. 2011 Nov 1;13(6):R109. doi: 10.1186/bcr3051 (PMC3326551; doi:10.1186/bcr3051)
Supplement: Additional file 3 — Table S3 (WORD file). Multivariate analysis of DFS according to histo-clinical variables. [file bcr3051-S3.DOC]

**Suppl Table 3 : Multivariate analysis of DFS according to histo-clinical variables.**

| **Variable** | | **HR for relapse** | **95%CI** | ***p*-value** |
| --- | --- | --- | --- | --- |
| Age | ≥ 50 years | 1 |  |  |
|  | < 50 years | 1.31 | 1.01 – 1.69 | 0.044 |
| Pathological tumor size (pT) | < 2 cm | 1 |  |  |
|  | ≥ 2 cm | 1.78 | 1.28 - 2.46 | <.001 |
| SBR Grade | I | 1 |  |  |
|  | II | 1.77 | 0.94 - 3.33 | 0.076 |
|  | III | 2.46 | 1.31 - 4.63 | 0.005 |
| Positive lymph nodes | 1 - 3 | 1 |  |  |
|  | > 3 | 1.94 | 1.49 - 2.53 | <.001 |
| Hormone receptors | Positive (ER and/or PR) | 1 |  |  |
|  | Negative (ER and PR) | 1.76 | 1.31 – 2.38 | <.001 |
| Chemotherapy arm | FEC | 1 |  |  |
|  | FEC-D | 0.78 | 0.60 - 1.02 | 0.066 |
