# Supplementary figures and images for: Protein expression, survival and docetaxel benefit in node-positive breast cancer treated with adjuvant chemotherapy in the FNCLCC - PACS 01 randomized trial
Source: Breast Cancer Res. 2011 Nov 1;13(6):R109. doi: 10.1186/bcr3051 (PMC3326551; doi:10.1186/bcr3051)

## Slide 1
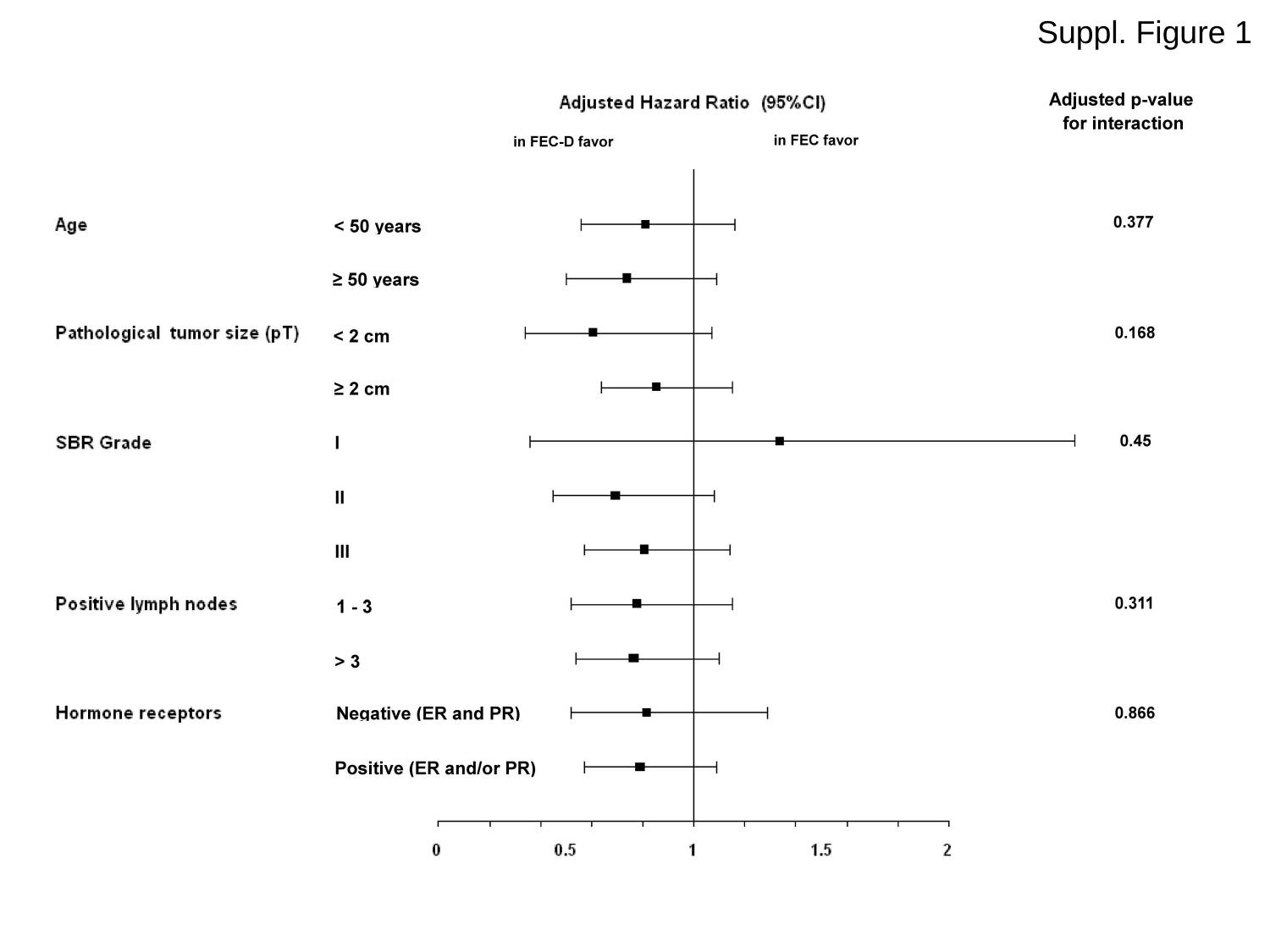

Suppl. Figure 1

Supplement: Additional file 4 — Figure S1 (POWER POINT file). Adjusted hazard ratios associated with docetaxel addition (forest plots) according to histo-clinical variables. [file bcr3051-S4.PPT]

## Slide 1
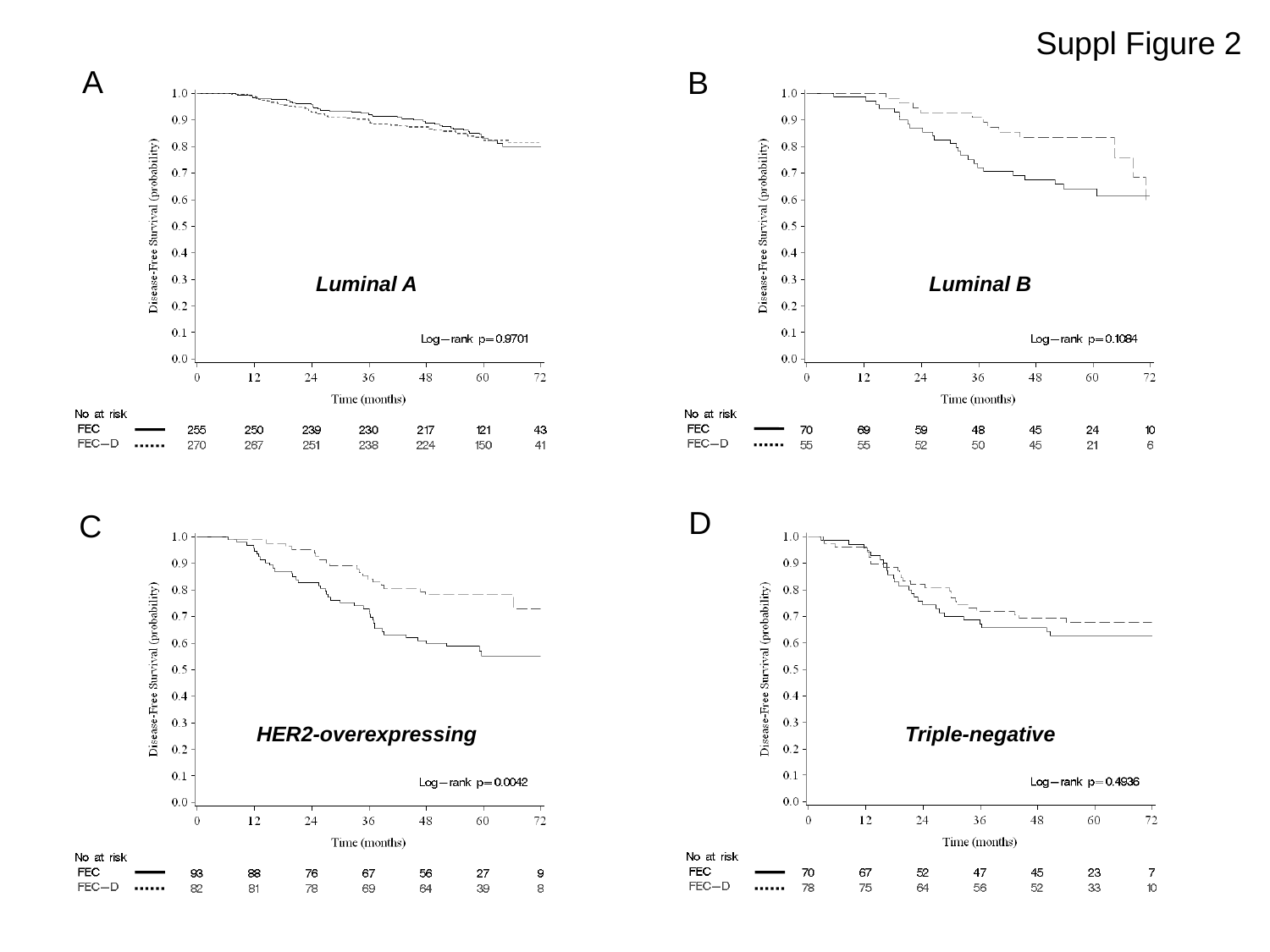

Suppl Figure 2
A
Luminal A
B
Luminal B
D
Triple-negative
C
HER2-overexpressing

Supplement: Additional file 7 — Figure S2 (POWER POINT file). Disease-free survival according to molecular subtypes and docetaxel. Kaplan-Meier DFS curves in patients with luminal A (A), luminal B (B), HER2-overexpressing (C) and triple-negative (D) tumor according to docetaxel addition. [file bcr3051-S7.PPT]
