# Supplementary material for: Protein expression, survival and docetaxel benefit in node-positive breast cancer treated with adjuvant chemotherapy in the FNCLCC - PACS 01 randomized trial
Source: Breast Cancer Res. 2011 Nov 1;13(6):R109. doi: 10.1186/bcr3051 (PMC3326551; doi:10.1186/bcr3051)
Supplement: Additional file 5 — Table S4 (WORD file). Univariate and multivariate analyses of 34 antibodies for interaction with chemotherapy arm. [file bcr3051-S5.DOC]

**²Suppl Table 4 :** **Univariate and multivariate analyses of 34 antibodies for interaction with chemotherapy arm**

| **Marker** | **Category** | **Treatment arm** | **N** | **Univariate** | | **Multivariate** | |
| --- | --- | --- | --- | --- | --- | --- | --- |
|  |  |  |  | **Unadjusted**  **Hazard Ratio**  **(95%CI)** | ***p*-value**  **for interaction** | **Adjusted**  **Hazard Ratio**  **(95%CI)** | ***p*-value**  **for interaction** |
| **AF6** | **Neg.** | FEC | 99 |  | 0.574 |  | 0.663 |
|  |  | FEC-D | 94 | 0.94 (0.54 - 1.66) |  | 0.94 (0.50 - 1.79) |  |
|  | **Pos.** | FEC | 321 |  |  |  |  |
|  |  | FEC-D | 335 | 0.78 (0.57 - 1.09) |  | 0.86 (0.61 - 1.23) |  |
| **Angiogenin** | **Neg.** | FEC | 34 |  | 0.653 |  | 0.100 |
|  |  | FEC-D | 37 | 0.85 (0.33 - 2.19) |  | 1.68 (0.47 - 5.96) |  |
|  | **Pos.** | FEC | 439 |  |  |  |  |
|  |  | FEC-D | 440 | 0.67 (0.51 - 0.88) |  | 0.66 (0.49 - 0.89) |  |
| **Aurora A** | **Neg.** | FEC | 293 |  | 0.074 |  | 0.228 |
|  |  | FEC-D | 292 | 0.87 (0.62 - 1.24) |  | 0.91 (0.62 - 1.33) |  |
|  | **Pos.** | FEC | 135 |  |  |  |  |
|  |  | FEC-D | 141 | 0.52 (0.33 - 0.82) |  | 0.61 (0.38 - 1.00) |  |
| **BCL2** | **Neg.** | FEC | 184 |  | 0.943 |  | 0.905 |
|  |  | FEC-D | 193 | 0.72 (0.50 - 1.03) |  | 0.78 (0.52 - 1.17) |  |
|  | **Pos.** | FEC | 285 |  |  |  |  |
|  |  | FEC-D | 295 | 0.70 (0.49 - 1.01) |  | 0.80 (0.54 - 1.19) |  |
| **α-Catenin** | **Neg.** | FEC | 177 |  | 0.927 |  | 0.875 |
|  |  | FEC-D | 182 | 0.77 (0.51 - 1.16) |  | 0.85 (0.53 - 1.37) |  |
|  | **Pos.** | FEC | 250 |  |  |  |  |
|  |  | FEC-D | 251 | 0.75 (0.52 - 1.09) |  | 0.79 (0.53 - 1.16) |  |
| **β-Catenin** | **Neg.** | FEC | 123 |  | 0.201 |  | 0.140 |
|  |  | FEC-D | 142 | 0.94 (0.59 - 1.49) |  | 1.19 (0.69 - 2.05) |  |
|  | **Pos.** | FEC | 324 |  |  |  |  |
|  |  | FEC-D | 312 | 0.65 (0.46 - 0.91) |  | 0.67 (0.47 - 0.96) |  |
| **CAV1** | **Neg.** | FEC | 82 |  | 0.343 |  | 0.229 |
|  |  | FEC-D | 97 | 0.97 (0.49 - 1.90) |  | 1.11 (0.50 - 2.44) |  |
|  | **Pos.** | FEC | 389 |  |  |  |  |
|  |  | FEC-D | 389 | 0.68 (0.51 - 0.90) |  | 0.69 (0.51 - 0.94) |  |
| **CD10** | **Neg.** | FEC | 197 |  | 0.574 |  | 0.741 |
|  |  | FEC-D | 212 | 0.81 (0.54 - 1.22) |  | 0.87 (0.55 - 1.38) |  |
|  | **Pos.** | FEC | 259 |  |  |  |  |
|  |  | FEC-D | 249 | 0.69 (0.49 - 0.98) |  | 0.77 (0.54 - 1.12) |  |
| **CD44** | **Neg.** | FEC | 210 |  | 0.292 |  | 0.158 |
|  |  | FEC-D | 220 | 0.68 (0.47 - 0.99) |  | 0.63 (0.41 - 0.96) |  |
|  | **Pos.** | FEC | 128 |  |  |  |  |
|  |  | FEC-D | 152 | 0.95 (0.58 - 1.56) |  | 1.03 (0.61 - 1.73) |  |
| **CK5/6** | **Neg.** | FEC | 122 |  | 0.914 |  | 0.960 |
|  |  | FEC-D | 126 | 0.76 (0.47 - 1.24) |  | 0.84 (0.48 - 1.48) |  |
|  | **Pos.** | FEC | 332 |  |  |  |  |
|  |  | FEC-D | 335 | 0.74 (0.54 - 1.01) |  | 0.79 (0.56 - 1.10) |  |
| **CK8/18** | **Neg.** | FEC | 13 |  | 0.041 |  | 0.446 |
|  |  | FEC-D | 9 | 2.22 (0.71 - 6.93) |  | 4.73 (0.96 - 23.3) |  |
|  | **Pos.** | FEC | 468 |  |  |  |  |
|  |  | FEC-D | 480 | 0.65 (0.50 - 0.85) |  | 0.69 (0.52 - 0.91) |  |
| **CK14** | **Neg.** | FEC | 386 |  | 0.506 |  | 0.142 |
|  |  | FEC-D | 388 | 0.71 (0.53 - 0.94) |  | 0.77 (0.56 - 1.05) |  |
|  | **Pos.** | FEC | 84 |  |  |  |  |
|  |  | FEC-D | 74 | 0.55 (0.28 - 1.07) |  | 0.41 (0.18 - 0.90) |  |
| **Cyclin D1** | **Neg.** | FEC | 161 |  | 0.775 |  | 0.974 |
|  |  | FEC-D | 155 | 0.71 (0.46 - 1.10) |  | 0.78 (0.48 - 1.26) |  |
|  | **Pos.** | FEC | 317 |  |  |  |  |
|  |  | FEC-D | 331 | 0.77 (0.56 - 1.06) |  | 0.78 (0.55 - 1.10) |  |
| **E-Cadherin** | **Neg.** | FEC | 62 |  | 0.507 |  | 0.154 |
|  |  | FEC-D | 66 | 0.90 (0.44 - 1.84) |  | 1.77 (0.69 - 4.59) |  |
|  | **Pos.** | FEC | 432 |  |  |  |  |
|  |  | FEC-D | 441 | 0.69 (0.53 - 0.91) |  | 0.71 (0.53 - 0.95) |  |
| **EGFR** | **Neg.** | FEC | 401 |  | 0.154 |  | 0.235 |
|  |  | FEC-D | 413 | 0.84 (0.64 - 1.11) |  | 0.85 (0.63 - 1.16) |  |
|  | **Pos.** | FEC | 95 |  |  |  |  |
|  |  | FEC-D | 90 | 0.52 (0.28 - 0.95) |  | 0.58 (0.30 – 1.13) |  |
| **ER** | **Neg.** | FEC | 137 |  | 0.438 |  | 0.976 |
|  |  | FEC-D | 146 | 0.63 (0.42 - 0.94) |  | 0.79 (0.52 - 1.22) |  |
|  | **Pos.** | FEC | 394 |  |  |  |  |
|  |  | FEC-D | 391 | 0.77 (0.57 - 1.05) |  | 0.79 (0.56 - 1.10) |  |
| **FGFR1** | **Neg.** | FEC | 56 |  | 0.092 |  | 0.178 |
|  |  | FEC-D | 58 | 0.34 (0.13 - 0.88) |  | 0.32 (0.10 - 1.02) |  |
|  | **Pos.** | FEC | 306 |  |  |  |  |
|  |  | FEC-D | 320 | 0.80 (0.58 - 1.11) |  | 0.84 (0.59 - 1.19) |  |
| **FHIT** | **Neg.** | FEC | 127 |  | 0.061 |  | 0.473 |
|  |  | FEC-D | 110 | 1.18 (0.70 - 2.00) |  | 0.00 (0.52 - 1.90) |  |
|  | **Pos.** | FEC | 321 |  |  |  |  |
|  |  | FEC-D | 351 | 0.66 (0.48 - 0.90) |  | 0.73 (0.52 - 1.01) |  |
| **GATA3** | **Neg.** | FEC | 88 |  | 0.702 |  | 0.908 |
|  |  | FEC-D | 78 | 0.80 (0.46 - 1.39) |  | 0.74 (0.40 - 1.37) |  |
|  | **Pos.** | FEC | 407 |  |  |  |  |
|  |  | FEC-D | 409 | 0.71 (0.53 - 0.94) |  | 0.74 (0.54 - 1.02) |  |
| **HER2** | **Neg.** | FEC | 451 |  | 0.054 |  | 0.367 |
|  |  | FEC-D | 466 | 0.84 (0.64 - 1.10) |  | 0.85 (0.63 - 1.15) |  |
|  | **Pos.** | FEC | 93 |  |  |  |  |
|  |  | FEC-D | 82 | 0.46 (0.27 - 0.79) |  | 0.65 (0.36 - 1.17) |  |
| **Ki67** | **Neg.** | FEC | 327 |  | 0.128 |  | 0.012 |
|  |  | FEC-D | 334 | 0.82 (0.59 - 1.16) |  | 1.10 (0.75 - 1.61) |  |
|  | **Pos.** | FEC | 147 |  |  |  |  |
|  |  | FEC-D | 133 | 0.54 (0.36 - 0.82) |  | 0.51 (0.33 - 0.79) |  |
| **MET** | **Neg.** | FEC | 301 |  | 0.128 |  | 0.154 |
|  |  | FEC-D | 301 | 0.85 (0.61 - 1.19) |  | 0.91 (0.63 - 1.30) |  |
|  | **Pos.** | FEC | 150 |  |  |  |  |
|  |  | FEC-D | 165 | 0.56 (0.36 - 0.86) |  | 0.59 (0.38 - 0.94) |  |
| **Moesin** | **Neg.** | FEC | 412 |  | 0.849 |  | 0.920 |
|  |  | FEC-D | 412 | 0.72 (0.54 - 0.96) |  | 0.77 (0.57 - 1.05) |  |
|  | **Pos.** | FEC | 56 |  |  |  |  |
|  |  | FEC-D | 62 | 0.77 (0.40 - 1.52) |  | 0.80 (0.39 - 1.65) |  |
| **MUC1** | **Neg.** | FEC | 53 |  | 0.726 |  | 0.651 |
|  |  | FEC-D | 42 | 0.82 (0.41 - 1.63) |  | 0.73 (0.32 - 1.70) |  |
|  | **Pos.** | FEC | 461 |  |  |  |  |
|  |  | FEC-D | 477 | 0.71 (0.55 - 0.93) |  | 0.73 (0.55 - 0.98) |  |
| **P21** | **Neg.** | FEC | 187 |  | 0.963 |  | 0.879 |
|  |  | FEC-D | 191 | 0.75 (0.49 - 1.13) |  | 0.74 (0.48 - 1.14) |  |
|  | **Pos.** | FEC | 272 |  |  |  |  |
|  |  | FEC-D | 273 | 0.74 (0.52 - 1.05) |  | 0.76 (0.51 - 1.13) |  |
| **P27** | **Neg.** | FEC | 78 |  | 0.737 |  | 0.826 |
|  |  | FEC-D | 97 | 0.66 (0.37 - 1.18) |  | 0.76 (0.41 - 1.42) |  |
|  | **Pos.** | FEC | 378 |  |  |  |  |
|  |  | FEC-D | 390 | 0.74 (0.55 - 0.99) |  | 0.73 (0.53 - 1.00) |  |
| **P53** | **Neg.** | FEC | 364 |  | 0.974 |  | 0.740 |
|  |  | FEC-D | 382 | 0.71 (0.52 - 0.96) |  | 0.76 (0.54 - 1.08) |  |
|  | **Pos.** | FEC | 123 |  |  |  |  |
|  |  | FEC-D | 123 | 0.70 (0.45 - 1.10) |  | 0.71 (0.45 - 1.13) |  |
| **P-Cadherin** | **Neg.** | FEC | 282 |  | 0.355 |  | 0.622 |
|  |  | FEC-D | 288 | 0.82 (0.58 - 1.17) |  | 0.78 (0.52 - 1.17) |  |
|  | **Pos.** | FEC | 179 |  |  |  |  |
|  |  | FEC-D | 192 | 0.64 (0.44 - 0.94) |  | 0.68 (0.46 - 1.02) |  |
| **PR** | **Neg.** | FEC | 255 |  | 0.228 |  | 0.601 |
|  |  | FEC-D | 233 | 0.85 (0.62 - 1.17) |  | 0.86 (0.61 - 1.22) |  |
|  | **Pos.** | FEC | 276 |  |  |  |  |
|  |  | FEC-D | 305 | 0.63 (0.43 - 0.92) |  | 0.76 (0.50 - 1.15) |  |
| **PTEN** | **Neg.** | FEC | 152 |  | 0.090 |  | 0.221 |
|  |  | FEC-D | 162 | 1.04 (0.66 - 1.64) |  | 0.99 (0.60 - 1.64) |  |
|  | **Pos.** | FEC | 315 |  |  |  |  |
|  |  | FEC-D | 295 | 0.64 (0.46 - 0.89) |  | 0.68 (0.47 - 0.97) |  |
| **TACC2** | **Neg.** | FEC | 70 |  | 0.980 |  | 0.397 |
|  |  | FEC-D | 78 | 0.77 (0.40 - 1.50) |  | 0.54 (0.25 - 1.20) |  |
|  | **Pos.** | FEC | 366 |  |  |  |  |
|  |  | FEC-D | 357 | 0.78 (0.58 - 1.05) |  | 0.87 (0.63 - 1.19) |  |
| **TACC3** | **Neg.** | FEC | 13 |  | 0.288 |  | 0.363 |
|  |  | FEC-D | 22 | 0.33 (0.05 - 1.97) |  | 0.29 (0.04 - 2.14) |  |
|  | **Pos.** | FEC | 284 |  |  |  |  |
|  |  | FEC-D | 280 | 0.88 (0.63 - 1.24) |  | 0.97 (0.67 - 1.41) |  |
| **TAU** | **Neg.** | FEC | 340 |  | 0.536 |  | 0.611 |
|  |  | FEC-D | 345 | 0.75 (0.55 - 1.01) |  | 0.83 (0.59 - 1.16) |  |
|  | **Pos.** | FEC | 65 |  |  |  |  |
|  |  | FEC-D | 76 | 0.99 (0.43 - 2.29) |  | 0.96 (0.38 - 2.42) |  |
| **TOPO2A** | **Neg.** | FEC | 104 |  | 0.109 |  | 0.917 |
|  |  | FEC-D | 95 | 1.20 (0.64 - 2.28) |  | 0.82 (0.39 - 1.74) |  |
|  | **Pos.** | FEC | 359 |  |  |  |  |
|  |  | FEC-D | 355 | 0.68 (0.50 - 0.91) |  | 0.77 (0.56 - 1.06) |  |
