# Supplementary material for: Protein expression, survival and docetaxel benefit in node-positive breast cancer treated with adjuvant chemotherapy in the FNCLCC - PACS 01 randomized trial
Source: Breast Cancer Res. 2011 Nov 1;13(6):R109. doi: 10.1186/bcr3051 (PMC3326551; doi:10.1186/bcr3051)
Supplement: Additional file 6 — Table S5 (WORD file). Molecular subtypes and correlations with histo-clinical features and IHC results. [file bcr3051-S6.DOC]

**Suppl. Table 5 : Molecular sub-types and correlations with histo-clinical features and IHC results.**

| **Characteristic** | **Luminal A**  **(N = 525)** | **Luminal B**  **(N = 125)** | **Triple-negative**  **(N = 148)** | **HER2-overexpressing**  **(N = 175)** | ***p*-value** |
| --- | --- | --- | --- | --- | --- |
| **Age** |  |  |  |  |  |
| < 50 years | 249 (47.4%) | 64 (51.2%) | 69 (46.6%) | 90 (51.4%) | 0.702 |
| ≥ 50 years | 276 (52.6%) | 61 (48.8%) | 79 (53.4%) | 85 (48.6%) |  |
| **Menopausal status** |  |  |  |  |  |
| Premenopausal | 202 (38.9%) | 38 (31.4%) | 53 (36.8%) | 64 (37%) | 0.493 |
| Postmenopausal | 317 (61.1%) | 83 (68.6%) | 91 (63.2%) | 109 (63%) |  |
| **Surgery** |  |  |  |  |  |
| Breast conservation | 311 (59.2%) | 80 (64%) | 96 (64.9%) | 95 (54.3%) | 0.189 |
| Modified mastectomy | 214 (40.8%) | 45 (36%) | 52 (35.1%) | 80 (45.7%) |  |
| **Pathological tumor size (pT)** |  |  |  |  |  |
| < 2 cm | 186 (38.4%) | 30 (25.9%) | 45 (33.6%) | 50 (31.1%) | 0.024 |
| 2 ≤ pT <-5 cm | 258 (53.3%) | 79 (68.1%) | 81 (60.5%) | 105 (65.2%) |  |
| ≥ 5 cm | 40 (8.3%) | 7 (6%) | 8 (6%) | 6 (3.7%) |  |
| **SBR Grade** |  |  |  |  |  |
| I | 87 (16.7%) | 3 (2.4%) | 7 (4.8%) | 5 (2.9%) | 0.000 |
| II | 274 (52.6%) | 54 (43.2%) | 30 (20.4%) | 47 (26.9%) |  |
| III | 132 (25.3%) | 64 (51.2%) | 104 (70.8%) | 118 (67.4%) |  |
| Not gradable | 28 (5.4%) | 4 (3.2%) | 6 (4.1%) | 5 (2.9%) |  |
| **Positive lymph nodes** |  |  |  |  |  |
| 1 - 3 | 319 (60.8%) | 73 (58.4%) | 102 (68.9%) | 90 (51.4%) | 0.015 |
| ≥ 4 | 206 (39.2%) | 52 (41.6%) | 46 (31.1%) | 85 (48.6%) |  |
| **Hormone receptors** |  |  |  |  |  |
| Negative (ER and PR) |  |  | 148 (100%) | 86 (51.5%) | 0.000 |
| Positive (ER and/or PR) | 525 (100%) | 125 (100%) |  | 81 (48.5%) |  |
| **AF6** |  |  |  |  |  |
| Negative | 85 (21.1%) | 12 (11.2%) | 24 (21.1%) | 27 (20%) | 0.137 |
| Positive | 318 (78.9%) | 95 (88.8%) | 90 (79%) | 108 (80%) |  |
| **Angiogenin** |  |  |  |  |  |
| Negative | 31 (6.9%) | 12 (10.7%) | 10 (7.8%) | 5 (3.3%) | 0.118 |
| Positive | 420 (93.1%) | 100 (89.3%) | 118 (92.2%) | 148 (96.7%) |  |
| **Aurora A** |  |  |  |  |  |
| Negative | 283 (70.6%) | 67 (62%) | 71 (59.7%) | 79 (56.8%) | 0.009 |
| Positive | 118 (29.4%) | 41 (38%) | 48 (40.3%) | 60 (43.2%) |  |
| **BCL2** |  |  |  |  |  |
| Negative | 105 (22.8%) | 40 (34.5%) | 91 (71.7%) | 105 (69.5%) | 0.000 |
| Positive | 355 (77.2%) | 76 (65.5%) | 36 (28.4%) | 46 (30.5%) |  |
| **α-Catenin** |  |  |  |  |  |
| Negative | 163 (40.6%) | 37 (33.9%) | 58 (47.9%) | 54 (39.1%) | 0.185 |
| Positive | 238 (59.4%) | 72 (66.1%) | 63 (52.1%) | 84 (60.9%) |  |
| **β-Catenin** |  |  |  |  |  |
| Negative | 124 (29.4%) | 22 (19.6%) | 37 (31.1%) | 43 (30.1%) | 0.168 |
| Positive | 298 (70.6%) | 90 (80.4%) | 82 (68.9%) | 100 (69.9%) |  |
| **CAV1** |  |  |  |  |  |
| Negative | 115 (25%) | 19 (16.2%) | 15 (11.7%) | 17 (11.4%) | 0.000 |
| Positive | 345 (75%) | 98 (83.8%) | 113 (88.3%) | 132 (88.6%) |  |
| **CD10** |  |  |  |  |  |
| Negative | 197 (46.5%) | 39 (35.5%) | 46 (36.2%) | 54 (35.8%) | 0.024 |
| Positive | 227 (53.5%) | 71 (64.6%) | 81 (63.8%) | 97 (64.2%) |  |
| **CD44** |  |  |  |  |  |
| Negative | 212 (64.6%) | 54 (61.4%) | 47 (54%) | 72 (57.1%) | 0.222 |
| Positive | 116 (35.4%) | 34 (38.6%) | 40 (46%) | 54 (42.9%) |  |
| **CK5/6** |  |  |  |  |  |
| Negative | 119 (27.3%) | 33 (30.3%) | 25 (19.7%) | 35 (24%) | 0.225 |
| Positive | 317 (72.7%) | 76 (69.7%) | 102 (80.3%) | 111 (76%) |  |
| **CK8/18** |  |  |  |  |  |
| Negative | 2 (0.4%) | 1 (0.9%) | 14 (10.9%) | 4 (2.6%) | 0.000 |
| Positive | 462 (99.6%) | 113 (99.1%) | 115 (89.2%) | 150 (97.4%) |  |
| **CK14** |  |  |  |  |  |
| Negative | 378 (86.1%) | 97 (84.4%) | 92 (71.3%) | 118 (79.7%) | 0.001 |
| Positive | 61 (13.9%) | 18 (15.7%) | 37 (28.7%) | 30 (20.3%) |  |
| **Cyclin D1** |  |  |  |  |  |
| Negative | 119 (26%) | 24 (20.7%) | 88 (66.7%) | 58 (37.9%) | 0.000 |
| Positive | 339 (74%) | 92 (79.3%) | 44 (33.3%) | 95 (62.1%) |  |
| **E-Cadherin** |  |  |  |  |  |
| Negative | 68 (14.1%) | 11 (9.5%) | 11 (8.4%) | 17 (10.8%) | 0.213 |
| Positive | 415 (85.9%) | 105 (90.5%) | 120 (91.6%) | 141 (89.2%) |  |
| **EGFR** |  |  |  |  |  |
| Negative | 438 (90.3%) | 105 (91.3%) | 62 (46.3%) | 108 (70.6%) | 0.000 |
| Positive | 47 (9.7%) | 10 (8.7%) | 72 (53.7%) | 45 (29.4%) |  |
| **ER** |  |  |  |  |  |
| Negative | 22 (4.2%) | 6 (4.8%) | 148 (100%) | 96 (57.5%) | 0.000 |
| Positive | 503 (95.8%) | 119 (95.2%) |  | 71 (42.5%) |  |
| **FGFR1** |  |  |  |  |  |
| Negative | 48 (13.9%) | 14 (14.1%) | 20 (19.8%) | 16 (14.4%) | 0.519 |
| Positive | 298 (86.1%) | 85 (85.9%) | 81 (80.2%) | 95 (85.6%) |  |
| **FHIT** |  |  |  |  |  |
| Negative | 98 (22.7%) | 28 (25.7%) | 40 (33.3%) | 39 (26.9%) | 0.124 |
| Positive | 333 (77.3%) | 81 (74.3%) | 80 (66.7%) | 106 (73.1%) |  |
| **GATA3** |  |  |  |  |  |
| Negative | 46 (9.8%) | 3 (2.5%) | 47 (35.9%) | 46 (29.9%) | 0.000 |
| Positive | 423 (90.2%) | 116 (97.5%) | 84 (64.1%) | 108 (70.1%) |  |
| **HER2** |  |  |  |  |  |
| Negative | 525 (100%) | 125 (100%) | 148 (100%) |  | 0.000 |
| Positive |  |  |  | 175 (100%) |  |
| **Ki67** |  |  |  |  |  |
| Negative | 525 (100%) |  | 49 (37.4%) | 77 (52%) | 0.000 |
| Positive |  | 125 (100%) | 82 (62.6%) | 71 (48%) |  |
| **MET** |  |  |  |  |  |
| Negative | 297 (68.1%) | 65 (58%) | 76 (61.3%) | 86 (59.7%) | 0.090 |
| Positive | 139 (31.9%) | 47 (42%) | 48 (38.7%) | 58 (40.3%) |  |
| **Moesin** |  |  |  |  |  |
| Negative | 414 (93%) | 106 (92.2%) | 80 (61.5%) | 123 (83.7%) | 0.000 |
| Positive | 31 (7%) | 9 (7.8%) | 50 (38.5%) | 24 (16.3%) |  |
| **MUC1** |  |  |  |  |  |
| Negative | 36 (7.3%) | 11 (9.2%) | 35 (24.7%) | 7 (4.4%) | 0.000 |
| Positive | 459 (92.7%) | 109 (90.8%) | 107 (75.4%) | 151 (95.6%) |  |
| **P21** |  |  |  |  |  |
| Negative | 166 (37.6%) | 29 (25.7%) | 72 (57.6%) | 63 (43.8%) | 0.000 |
| Positive | 275 (62.4%) | 84 (74.3%) | 53 (42.4%) | 81 (56.3%) |  |
| **P27** |  |  |  |  |  |
| Negative | 58 (13.1%) | 23 (20.5%) | 39 (30.5%) | 39 (25.5%) | 0.000 |
| Positive | 386 (86.9%) | 89 (79.5%) | 89 (69.5%) | 114 (74.5%) |  |
| **P53** |  |  |  |  |  |
| Negative | 404 (86.3%) | 84 (72.4%) | 69 (48.9%) | 93 (58.9%) | 0.000 |
| Positive | 64 (13.7%) | 32 (27.6%) | 72 (51.1%) | 65 (41.1%) |  |
| **P-Cadherin** |  |  |  |  |  |
| Negative | 331 (74.6%) | 69 (60%) | 35 (26.9%) | 61 (40.7%) | 0.000 |
| Positive | 113 (25.5%) | 46 (40%) | 95 (73.1%) | 89 (59.3%) |  |
| **PR** |  |  |  |  |  |
| Negative | 148 (28.2%) | 40 (32%) | 148 (100%) | 113 (67.3%) | 0.000 |
| Positive | 377 (71.8%) | 85 (68%) |  | 55 (32.7%) |  |
| **PTEN** |  |  |  |  |  |
| Negative | 141 (32.6%) | 52 (45.6%) | 49 (38%) | 36 (24.7%) | 0.003 |
| Positive | 291 (67.4%) | 62 (54.4%) | 80 (62%) | 110 (75.3%) |  |
| **TACC2** |  |  |  |  |  |
| Negative | 57 (14%) | 17 (15.9%) | 20 (16.5%) | 16 (11.5%) | 0.654 |
| Positive | 350 (86%) | 90 (84.1%) | 101 (83.5%) | 123 (88.5%) |  |
| **TACC3** |  |  |  |  |  |
| Negative | 12 (4.4%) | 4 (4.9%) | 6 (7.3%) | 3 (3%) | 0.585 |
| Positive | 260 (95.6%) | 77 (95.1%) | 76 (92.7%) | 96 (97%) |  |
| **TAU** |  |  |  |  |  |
| Negative | 303 (76.1%) | 80 (83.3%) | 111 (97.4%) | 116 (94.3%) | 0.000 |
| Positive | 95 (23.9%) | 16 (16.7%) | 3 (2.6%) | 7 (5.7%) |  |
| **TOPO2A** |  |  |  |  |  |
| Negative | 108 (25%) | 4 (3.6%) | 29 (23.4%) | 23 (15.7%) | 0.000 |
| Positive | 324 (75%) | 108 (96.4%) | 95 (76.6%) | 124 (84.4%) |  |
