# Supplementary material for: Protein expression, survival and docetaxel benefit in node-positive breast cancer treated with adjuvant chemotherapy in the FNCLCC - PACS 01 randomized trial
Source: Breast Cancer Res. 2011 Nov 1;13(6):R109. doi: 10.1186/bcr3051 (PMC3326551; doi:10.1186/bcr3051)
Supplement: Additional file 8 — Table S6 (WORD file). Univariate and multivariate analyses of molecular subtypes for interaction with chemotherapy arm. [file bcr3051-S8.DOC]

**Suppl. Table 6: Univariate and multivariate analyses of molecular subtypes for interaction with chemotherapy arm.**

| **Subtype** | **Treatment arm** | **N** | **Event** | **Univariate** | | | **Multivariate** | | |
| --- | --- | --- | --- | --- | --- | --- | --- | --- | --- |
|  |  |  |  | **Unadjusted**  **Hazard Ratio**  **95%CI** | ***p*-value^†^** | ***p*-value for interaction**  **^††^** | **Adjusted**  **Hazard Ratio**  **95%CI** | ***p*-value^†^** | ***p*-value for interaction**  **^††^** |
| **Luminal A** | FEC | 255 | 17% |  |  |  |  |  |  |
|  | FEC-D | 270 | 17% | 1.01  (0.66 - 1.53) | 0.970 |  | 1.16  (0.73 - 1.84) | 0.520 |  |
| **Luminal B** | FEC | 70 | 36% |  |  |  |  |  |  |
|  | FEC-D | 55 | 24% | 0.58  (0.30 - 1.14) | 0.113 | 0.172 | 0.47  (0.22 - 1.01) | 0.054 | 0.047 |
| **HER2-overpressing** | FEC | 93 | 43% |  |  |  |  |  |  |
|  | FEC-D | 82 | 23% | 0.46  (0.27 - 0.79) | 0.005 | 0.025 | 0.66  (0.37 - 1.19) | 0.167 | 0.139 |
| **Triple-negative** | FEC | 70 | 37% |  |  |  |  |  |  |
|  | FEC-D | 78 | 32% | 0.83  (0.48 - 1.43) | 0.494 | 0.571 | 0.88  (0.49 - 1.57) | 0.668 | 0.462 |

^†^ p-value for rejecting the hypothesis of no treatment effect in specific therapeutic subgroup (luminal A, luminal B, triple-negative, and HER-overexpressing).

^††^ p-value for rejecting the hypothesis of an homogeneous treatment effect in treatment between subgroups: HER2-overexpressing and luminal A, or triple-negative and luminal A, or luminal B and luminal A.
