# Supplementary material for: Protein expression, survival and docetaxel benefit in node-positive breast cancer treated with adjuvant chemotherapy in the FNCLCC - PACS 01 randomized trial
Source: Breast Cancer Res. 2011 Nov 1;13(6):R109. doi: 10.1186/bcr3051 (PMC3326551; doi:10.1186/bcr3051)
Supplement: Additional file 9 — Table S7 (WORD file). Univariate and multivariate analyses of molecular subtypes for interaction with chemotherapy arm, using another definition for luminal B and HER2-overexpressing subtypes. [file bcr3051-S9.DOC]

**Suppl. Table 7: Univariate and multivariate analyses of molecular subtypes for interaction with chemotherapy arm, using another definition for luminal B and HER2-overexpressing subtypes.**

| **Subtype** | **Treatment arm** | **N** | **Event** | **Univariate** | | | **Multivariate^†^** | | |
| --- | --- | --- | --- | --- | --- | --- | --- | --- | --- |
|  |  |  |  | **Unadjusted**  **Hazard Ratio**  **95%CI** | ***p*-value ^†^** | ***p*-value for interaction**  **^††^** | **Adjusted**  **Hazard Ratio**  **95%CI** | ***p*-value**  **^†^** | ***p*-value for interaction**  **^††^** |
| **Luminal A** | FEC | 255 | 17% |  |  |  |  |  |  |
|  | FEC-D | 270 | 17% | 1.01  (0.66 - 1.53) | 0.970 |  | 1.16  (0.73 - 1.84) | 0.520 |  |
| **Luminal B** | FEC | 114 | 37% |  |  |  |  |  |  |
|  | FEC-D | 92 | 23% | 0.54  (0.32 – 0.92) | 0.020 | 0.069 | 0.54  (0.30 – 0.97) | 0.038 | 0.042 |
| **HER2-overexpressing** | FEC | 46 | 48% |  |  |  |  |  |  |
|  | FEC-D | 40 | 28% | 0.49  (0.24 – 1.01) | 0.049 | 0.090 | 0.69  (0.32 - 1.46) | 0.327 | 0.239 |
| **Triple-negative** | FEC | 70 | 37% |  |  |  |  |  |  |
|  | FEC-D | 78 | 32% | 0.83  (0.48 - 1.43) | 0.494 | 0.571 | 0.88  (0.49 - 1.57) | 0.668 | 0.462 |

^†^  p-value for rejecting the hypothesis of no treatment effect in specific therapeutic subgroup (luminal A, luminal B, triple-negative, and HER-overexpressing).

^††^ p-value for rejecting the hypothesis of an homogeneous treatment effect in treatment between subgroups: HER2-overexpressing and luminal A, or triple-negative and luminal A, or luminal B and luminal A.
