# Supplementary material for: Protein expression, survival and docetaxel benefit in node-positive breast cancer treated with adjuvant chemotherapy in the FNCLCC - PACS 01 randomized trial
Source: Breast Cancer Res. 2011 Nov 1;13(6):R109. doi: 10.1186/bcr3051 (PMC3326551; doi:10.1186/bcr3051)
Supplement: Additional file 10 — Table S8 (WORD file). Univariate analyses of 34 antibodies for DFS per subtype. [file bcr3051-S10.DOC]

**Suppl. Table 8 : Univariate analyses of 34 antibodies for DFS per subtype**

|  |  | **Luminal A** | | | | **Luminal B** | | | | **Triple-negative** | | | | | **HER2-overexpressing** | | | |
| --- | --- | --- | --- | --- | --- | --- | --- | --- | --- | --- | --- | --- | --- | --- | --- | --- | --- | --- |
| *Marker -*  *Category* | | *Total (N)* | *Event* | *Risk Ratio 95%CI* | *p value** | *Total (N)* | *Event* | *Risk Ratio 95%CI* | *p value** | *Total (N)* | *Event* | *Risk Ratio 95%CI* | *p value** | *Total (N)* | | *Event* | *Risk Ratio 95%CI* | *p value** |
| AF6 | Neg. | 85 | 15 (18%) |  |  | 12 | 6 (50%) |  |  | 24 | 9 (38%) |  |  | 27 | | 7 (26%) |  |  |
|  | Pos. | 318 | 49 (15%) | .869 (0.49,1.55) | 0.634 | 95 | 29 (31%) | .579 (0.24,1.40) | 0.219 | 90 | 25 (28%) | .711 (0.33,1.52) | 0.378 | 108 | | 33 (31%) | 1.21 (0.54,2.74) | 0.644 |
| Angiogenin | Neg. | 31 | 8 (26%) |  |  | 12 | 4 (33%) |  |  | 10 | 3 (30%) |  |  | 5 | | 1 (20%) |  |  |
|  | Pos. | 420 | 69 (16%) | .623 (0.30,1.30) | 0.201 | 100 | 32 (32%) | 1.03 (0.36,2.92) | 0.956 | 118 | 39 (33%) | 1.20 (0.37,3.88) | 0.761 | 148 | | 50 (34%) | 2.03 (0.28,14.7) | 0.475 |
| Aurora A | Neg. | 283 | 47 (17%) |  |  | 67 | 23 (34%) |  |  | 71 | 18 (25%) |  |  | 79 | | 20 (25%) |  |  |
|  | Pos. | 118 | 24 (20%) | 1.23 (0.75,2.01) | 0.415 | 41 | 11 (27%) | .829 (0.40,1.71) | 0.611 | 48 | 21 (44%) | 1.91 (1.02,3.58) | 0.041 | 60 | | 23 (38%) | 1.66 (0.91,3.03) | 0.093 |
| BCL2 | Neg. | 105 | 26 (25%) |  |  | 40 | 16 (40%) |  |  | 91 | 29 (32%) |  |  | 105 | | 35 (33%) |  |  |
|  | Pos. | 355 | 53 (15%) | .549 (0.34,0.88) | 0.011 | 76 | 21 (28%) | .528 (0.27,1.02) | 0.055 | 36 | 13 (36%) | 1.22 (0.63,2.35) | 0.552 | 46 | | 14 (30%) | .896 (0.48,1.66) | 0.727 |
| **α**-Catenin | Neg. | 163 | 29 (18%) |  |  | 37 | 11 (30%) |  |  | 58 | 20 (34%) |  |  | 54 | | 18 (33%) |  |  |
|  | Pos. | 238 | 38 (16%) | .951 (0.59,1.54) | 0.838 | 72 | 25 (35%) | 1.23 (0.61,2.50) | 0.565 | 63 | 17 (27%) | .752 (0.39,1.43) | 0.385 | 84 | | 26 (31%) | .889 (0.49,1.62) | 0.701 |
| **β**-Catenin | Neg. | 124 | 24 (19%) |  |  | 22 | 8 (36%) |  |  | 37 | 14 (38%) |  |  | 43 | | 14 (33%) |  |  |
|  | Pos. | 298 | 47 (16%) | .820 (0.50,1.34) | 0.43 | 90 | 29 (32%) | .876 (0.40,1.92) | 0.741 | 82 | 24 (29%) | .758 (0.39,1.47) | 0.409 | 100 | | 31 (31%) | 1.00 (0.53,1.88) | 0.996 |
| CAV1 | Neg. | 115 | 19 (17%) |  |  | 19 | 4 (21%) |  |  | 15 | 5 (33%) |  |  | 17 | | 5 (29%) |  |  |
|  | Pos. | 345 | 61 (18%) | 1.07 (0.64,1.79) | 0.795 | 98 | 34 (35%) | 1.71 (0.61,4.85) | 0.303 | 113 | 36 (32%) | .982 (0.39,2.50) | 0.969 | 132 | | 43 (33%) | 1.00 (0.40,2.54) | 0.993 |
| CD10 | Neg. | 197 | 29 (15%) |  |  | 39 | 16 (41%) |  |  | 46 | 13 (28%) |  |  | 54 | | 18 (33%) |  |  |
|  | Pos. | 227 | 41 (18%) | 1.24 (0.77,2.00) | 0.371 | 71 | 20 (28%) | .637 (0.33,1.23) | 0.175 | 81 | 29 (36%) | 1.30 (0.68,2.50) | 0.428 | 97 | | 31 (32%) | .991 (0.55,1.77) | 0.976 |
| CD44 | Neg. | 212 | 41 (19%) |  |  | 54 | 21 (39%) |  |  | 47 | 17 (36%) |  |  | 72 | | 22 (31%) |  |  |
|  | Pos. | 116 | 16 (14%) | .701 (0.39,1.25) | 0.225 | 34 | 11 (32%) | .853 (0.41,1.77) | 0.67 | 40 | 13 (33%) | .882 (0.43,1.82) | 0.732 | 54 | | 15 (28%) | .821 (0.42,1.59) | 0.558 |
| CK14 | Neg. | 378 | 61 (16%) |  |  | 97 | 34 (35%) |  |  | 92 | 32 (35%) |  |  | 118 | | 40 (34%) |  |  |
|  | Pos. | 61 | 11 (18%) | 1.13 (0.59,2.14) | 0.714 | 18 | 3 (17%) | .390 (0.12,1.27) | 0.106 | 37 | 12 (32%) | .960 (0.49,1.86) | 0.902 | 30 | | 9 (30%) | .806 (0.39,1.66) | 0.558 |
| CK5/6 | Neg. | 119 | 24 (20%) |  |  | 33 | 9 (27%) |  |  | 25 | 10 (40%) |  |  | 35 | | 14 (40%) |  |  |
|  | Pos. | 317 | 49 (15%) | .758 (0.46,1.24) | 0.266 | 76 | 26 (34%) | 1.26 (0.59,2.68) | 0.556 | 102 | 33 (32%) | .773 (0.38,1.57) | 0.475 | 111 | | 34 (31%) | .763 (0.41,1.42) | 0.394 |
| CK8/18 | Neg. | 2 | 2 (100%) |  |  | 1 | 1 (100%) |  |  | 14 | 5 (36%) |  |  | 4 | | 3 (75%) |  |  |
|  | Pos. | 462 | 78 (17%) | .064 (0.02,0.27) | <.001 | 113 | 34 (30%) | .018 (0.00,0.20) | <.001 | 115 | 40 (35%) | .919 (0.36,2.33) | 0.859 | 150 | | 48 (32%) | .349 (0.11,1.12) | 0.065 |
| Cyclin D1 | Neg. | 119 | 16 (13%) |  |  | 24 | 8 (33%) |  |  | 88 | 28 (32%) |  |  | 58 | | 23 (40%) |  |  |
|  | Pos. | 339 | 58 (17%) | 1.33 (0.77,2.32) | 0.307 | 92 | 30 (33%) | .961 (0.44,2.10) | 0.921 | 44 | 17 (39%) | 1.27 (0.70,2.32) | 0.433 | 95 | | 28 (29%) | .670 (0.39,1.16) | 0.153 |
| E-Cadherin | Neg. | 68 | 12 (18%) |  |  | 11 | 3 (27%) |  |  | 11 | 3 (27%) |  |  | 17 | | 7 (41%) |  |  |
|  | Pos. | 415 | 69 (17%) | 1.00 (0.54,1.86) | 0.989 | 105 | 33 (31%) | 1.38 (0.42,4.52) | 0.592 | 120 | 40 (33%) | 1.27 (0.39,4.09) | 0.693 | 141 | | 44 (31%) | .792 (0.36,1.76) | 0.566 |
| EGFR | Neg. | 438 | 80 (18%) |  |  | 105 | 32 (30%) |  |  | 62 | 27 (44%) |  |  | 108 | | 36 (33%) |  |  |
|  | Pos. | 47 | 3 (6%) | .327 (0.10,1.04) | 0.045 | 10 | 4 (40%) | 1.35 (0.48,3.85) | 0.569 | 72 | 20 (28%) | .567 (0.32,1.01) | 0.051 | 45 | | 15 (33%) | 1.03 (0.56,1.88) | 0.925 |
| ER | Neg. | 22 | 2 (9%) |  |  | 6 | 4 (67%) |  |  | 148 | 51 (34%) | . ( . , . ) |  | 96 | | 35 (36%) |  |  |
|  | Pos. | 503 | 87 (17%) | 1.96 (0.48,7.98) | 0.337 | 119 | 34 (29%) | .339 (0.12,0.96) | 0.032 |  |  |  |  | 71 | | 23 (32%) | .812 (0.48,1.37) | 0.437 |
| FGFR1 | Neg. | 48 | 6 (13%) |  |  | 14 | 4 (29%) |  |  | 20 | 5 (25%) |  |  | 16 | | 2 (13%) |  |  |
|  | Pos. | 298 | 45 (15%) | 1.25 (0.53,2.93) | 0.609 | 85 | 28 (33%) | 1.15 (0.40,3.27) | 0.798 | 81 | 26 (32%) | 1.35 (0.52,3.51) | 0.539 | 95 | | 31 (33%) | 3.18 (0.76,13.3) | 0.094 |
| FHIT | Neg. | 98 | 18 (18%) |  |  | 28 | 6 (21%) |  |  | 40 | 11 (28%) |  |  | 39 | | 10 (26%) |  |  |
|  | Pos. | 333 | 57 (17%) | .950 (0.56,1.62) | 0.851 | 81 | 29 (36%) | 1.68 (0.70,4.05) | 0.242 | 80 | 28 (35%) | 1.33 (0.66,2.66) | 0.426 | 106 | | 35 (33%) | 1.31 (0.65,2.65) | 0.446 |
| GATA3 | Neg. | 46 | 8 (17%) |  |  | 3 | 2 (67%) |  |  | 47 | 20 (43%) |  |  | 46 | | 17 (37%) |  |  |
|  | Pos. | 423 | 72 (17%) | .962 (0.46,2.00) | 0.919 | 116 | 36 (31%) | .339 (0.08,1.42) | 0.121 | 84 | 24 (29%) | .636 (0.35,1.15) | 0.132 | 108 | | 33 (31%) | .793 (0.44,1.42) | 0.437 |
| HER2 | Neg. | 525 | 89 (17%) | . ( . , . ) |  | 125 | 38 (30%) | . ( . , . ) |  | 148 | 51 (34%) | . ( . , . ) |  |  | |  |  |  |
|  | Pos |  |  |  |  |  |  |  |  |  |  |  |  | 175 | | 59 (34%) | . ( . , . ) |  |
| Ki67 | Neg. | 525 | 89 (17%) | . ( . , . ) |  |  |  |  |  | 49 | 15 (31%) |  |  | 77 | | 27 (35%) |  |  |
|  | Pos. |  |  |  |  | 125 | 38 (30%) | . ( . , . ) |  | 82 | 31 (38%) | 1.30 (0.70,2.41) | 0.402 | 71 | | 27 (38%) | 1.12 (0.66,1.91) | 0.682 |
| MET | Neg. | 297 | 48 (16%) |  |  | 65 | 23 (35%) |  |  | 76 | 26 (34%) |  |  | 86 | | 25 (29%) |  |  |
|  | Pos. | 139 | 29 (21%) | 1.41 (0.89,2.23) | 0.145 | 47 | 13 (28%) | .759 (0.38,1.50) | 0.426 | 48 | 15 (31%) | .880 (0.47,1.66) | 0.692 | 58 | | 21 (36%) | 1.32 (0.74,2.37) | 0.341 |
| Moesin | Neg. | 414 | 65 (16%) |  |  | 106 | 32 (30%) |  |  | 80 | 35 (44%) |  |  | 123 | | 38 (31%) |  |  |
|  | Pos. | 31 | 9 (29%) | 2.19 (1.09,4.41) | 0.024 | 9 | 5 (56%) | 2.34 (0.91,6.02) | 0.069 | 50 | 10 (20%) | .379 (0.19,0.77) | 0.005 | 24 | | 9 (38%) | 1.24 (0.60,2.57) | 0.557 |
| MUC1 | Neg. | 36 | 10 (28%) |  |  | 11 | 4 (36%) |  |  | 35 | 14 (40%) |  |  | 7 | | 3 (43%) |  |  |
|  | Pos. | 459 | 73 (16%) | .570 (0.29,1.11) | 0.093 | 109 | 34 (31%) | .842 (0.30,2.38) | 0.745 | 107 | 36 (34%) | .786 (0.42,1.46) | 0.445 | 151 | | 49 (32%) | .766 (0.24,2.46) | 0.655 |
| P21 | Neg. | 166 | 27 (16%) |  |  | 29 | 9 (31%) |  |  | 72 | 24 (33%) |  |  | 63 | | 21 (33%) |  |  |
|  | Pos. | 275 | 46 (17%) | 1.06 (0.66,1.71) | 0.803 | 84 | 28 (33%) | .966 (0.46,2.05) | 0.93 | 53 | 18 (34%) | 1.07 (0.58,1.97) | 0.828 | 81 | | 23 (28%) | .821 (0.45,1.48) | 0.513 |
| P27 | Neg. | 58 | 11 (19%) |  |  | 23 | 7 (30%) |  |  | 39 | 11 (28%) |  |  | 39 | | 12 (31%) |  |  |
|  | Pos. | 386 | 63 (16%) | .884 (0.46,1.68) | 0.708 | 89 | 28 (31%) | 1.04 (0.45,2.38) | 0.929 | 89 | 31 (35%) | 1.30 (0.65,2.59) | 0.449 | 114 | | 39 (34%) | 1.21 (0.63,2.31) | 0.566 |
| P53 | Neg. | 404 | 63 (16%) |  |  | 84 | 27 (32%) |  |  | 69 | 27 (39%) |  |  | 93 | | 28 (30%) |  |  |
|  | Pos. | 64 | 16 (25%) | 1.67 (0.96,2.89) | 0.065 | 32 | 9 (28%) | .838 (0.39,1.78) | 0.645 | 72 | 22 (31%) | .708 (0.40,1.24) | 0.227 | 65 | | 24 (37%) | 1.30 (0.75,2.25) | 0.343 |
| P-Cadherin | Neg. | 331 | 58 (18%) |  |  | 69 | 18 (26%) |  |  | 35 | 16 (46%) |  |  | 61 | | 15 (25%) |  |  |
|  | Pos. | 113 | 20 (18%) | 1.03 (0.62,1.71) | 0.91 | 46 | 19 (41%) | 1.58 (0.83,3.02) | 0.16 | 95 | 29 (31%) | .568 (0.31,1.05) | 0.066 | 89 | | 32 (36%) | 1.59 (0.86,2.94) | 0.133 |
| PR | Neg. | 148 | 30 (20%) |  |  | 40 | 16 (40%) |  |  | 148 | 51 (34%) | . ( . , . ) |  | 113 | | 42 (37%) |  |  |
|  | Pos. | 377 | 59 (16%) | .783 (0.50,1.22) | 0.275 | 85 | 22 (26%) | .538 (0.28,1.03) | 0.056 |  |  |  |  | 55 | | 16 (29%) | .736 (0.41,1.31) | 0.296 |
| PTEN | Neg. | 141 | 22 (16%) |  |  | 52 | 20 (38%) |  |  | 49 | 16 (33%) |  |  | 36 | | 9 (25%) |  |  |
|  | Pos. | 291 | 51 (18%) | 1.12 (0.68,1.85) | 0.646 | 62 | 16 (26%) | .665 (0.34,1.29) | 0.224 | 80 | 27 (34%) | 1.05 (0.57,1.95) | 0.876 | 110 | | 36 (33%) | 1.41 (0.68,2.93) | 0.355 |
| TACC2 | Neg. | 57 | 10 (18%) |  |  | 17 | 4 (24%) |  |  | 20 | 12 (60%) |  |  | 16 | | 1 (6%) |  |  |
|  | Pos. | 350 | 62 (18%) | 1.02 (0.52,1.99) | 0.952 | 90 | 30 (33%) | 1.66 (0.58,4.71) | 0.338 | 101 | 30 (30%) | .368 (0.19,0.72) | 0.002 | 123 | | 42 (34%) | 6.41 (0.88,46.6) | 0.035 |
| TACC3 | Neg. | 12 | 1 (8%) |  |  | 4 | 1 (25%) |  |  | 6 | 1 (17%) |  |  | 3 | | 0 (0%) |  |  |
|  | Pos. | 260 | 43 (17%) | 2.06 (0.28,15.0) | 0.464 | 77 | 27 (35%) | 1.40 (0.19,10.3) | 0.739 | 76 | 22 (29%) | 1.81 (0.24,13.4) | 0.555 | 96 | | 28 (29%) | 13E5 (0.00, . ) | 0.305 |
| TAU | Neg. | 303 | 60 (20%) |  |  | 80 | 23 (29%) |  |  | 111 | 37 (33%) |  |  | 116 | | 37 (32%) |  |  |
|  | Pos. | 95 | 8 (8%) | .390 (0.19,0.82) | 0.01 | 16 | 6 (38%) | 1.34 (0.54,3.29) | 0.528 | 3 | 1 (33%) | .886 (0.12,6.46) | 0.904 | 7 | | 3 (43%) | 1.31 (0.40,4.27) | 0.649 |
| TOPO2A | Neg. | 108 | 12 (11%) |  |  | 4 | 0 (0%) |  |  | 29 | 11 (38%) |  |  | 23 | | 7 (30%) |  |  |
|  | Pos. | 324 | 61 (19%) | 1.74 (0.94,3.23) | 0.076 | 108 | 38 (35%) | 13E5 (0.00, . ) | 0.224 | 95 | 30 (32%) | .805 (0.40,1.61) | 0.537 | 124 | | 39 (31%) | 1.08 (0.48,2.41) | 0.858 |

*, log-rank test
