# Supplementary material for: Protein expression, survival and docetaxel benefit in node-positive breast cancer treated with adjuvant chemotherapy in the FNCLCC - PACS 01 randomized trial
Source: Breast Cancer Res. 2011 Nov 1;13(6):R109. doi: 10.1186/bcr3051 (PMC3326551; doi:10.1186/bcr3051)
Supplement: Additional file 11 — Table S9 (WORD file). Univariate and multivariate analyses of molecular subtypes for interaction with chemotherapy arm, using 15% cut-off for Ki67 for the definition of luminal subtypes. [file bcr3051-S11.DOC]

**Suppl. Table 9 : Univariate and multivariate analyses of molecular subtypes for interaction with chemotherapy arm, using 15% cut-off for Ki67 for the definition of luminal subtypes.**

| **Subtype** | **Treatment arm** | **N** | **Event** | **Univariate** | | | **Multivariate** | | |
| --- | --- | --- | --- | --- | --- | --- | --- | --- | --- |
|  |  |  |  | **Unadjusted**  **Hazard Ratio**  **95%CI** | ***p*-value^†^** | ***p*-value for interaction**  **^††^** | **Adjusted**  **Hazard Ratio**  **95%CI** | ***p*-value^†^** | ***p*-value for interaction**  **^††^** |
| **Luminal A** | FEC | 217 | 17% |  |  |  |  |  |  |
|  | FEC-D | 240 | 16% | .944  (0.60,1.48) | 0.801 |  | 1.11  (0.68,1.83) | 0.680 |  |
| **Luminal B** | FEC | 108 | 29% |  |  |  |  |  |  |
|  | FEC-D | 85 | 24% | .782  (0.45,1.37) | 0.390 | 0.608 | .665  (0.36,1.24) | 0.201 | 0.208 |
| **HER2-overpressing** | FEC | 93 | 43% |  |  |  |  |  |  |
|  | FEC-D | 82 | 23% | .459  (0.27,0.79) | 0.004 | 0.046 | .666  (0.37,1.19) | 0.168 | 0.188 |
| **Triple-negative** | FEC | 70 | 37% |  |  |  |  |  |  |
|  | FEC-D | 78 | 32% | .826  (0.48,1.43) | 0.494 | 0.712 | .882  (0.49,1.57) | 0.669 | 0.554 |

^†^ p-value for rejecting the hypothesis of no treatment effect in specific therapeutic subgroup (luminal A, luminal B, triple-negative, and HER-overexpressing).

^††^ p-value for rejecting the hypothesis of an homogeneous treatment effect in treatment between subgroups: HER2-overexpressing and luminal A, or triple-negative and luminal A, or luminal B and luminal A.
