# Supplementary material for: Protein expression, survival and docetaxel benefit in node-positive breast cancer treated with adjuvant chemotherapy in the FNCLCC - PACS 01 randomized trial
Source: Breast Cancer Res. 2011 Nov 1;13(6):R109. doi: 10.1186/bcr3051 (PMC3326551; doi:10.1186/bcr3051)
Supplement: Additional file 12 — Table S10 (WORD file). Range and quartiles used for each antibody. [file bcr3051-S12.DOC]

**Suppl. Table 10 : Range and quartiles used for each antibody.**

|  | **Percentiles** | | | | |
| --- | --- | --- | --- | --- | --- |
| **Marker** | **Min** | **Max** | **P25** | **P50 (Med)** | **P75** |
| AF6 | 0 | 300 | 15 | 80 | 120 |
| Angiogenin | 0 | 300 | 70 | 103 | 187 |
| Aurora A | 0 | 200 | 0 | 0 | 10 |
| BCL2 | 0 | 300 | 0 | 20 | 95 |
| α-Catenin | 0 | 200 | 0 | 10 | 50 |
| β-Catenin | 0 | 300 | 0 | 60 | 170 |
| CAV1 | 0 | 300 | 25 | 95 | 157 |
| CD10 | 0 | 300 | 0 | 35 | 100 |
| CD44 | 0 | 300 | 0 | 0 | 93.3 |
| CK14 | 0 | 270 | 0 | 0 | 0 |
| CK5/6 | 0 | 300 | 0 | 60 | 120 |
| CK8/18 | 0 | 300 | 250 | 300 | 300 |
| Cyclin D1 | 0 | 300 | 0 | 25 | 93.3 |
| E-Cadherin | 0 | 300 | 70 | 170 | 250 |
| EGFR | 0 | 300 | 0 | 0 | 0 |
| ER* | 0 | 100 | 0 | 60 | 80 |
| FGFR1 | 0 | 300 | 34.2 | 122 | 180 |
| FHIT | 0 | 290 | 0 | 40 | 86.7 |
| GATA3 | 0 | 300 | 16.7 | 100 | 183 |
| Ki67* | 0 | 90 | 1 | 10 | 30 |
| MET | 0 | 300 | 0 | 0 | 25 |
| Moesin | 0 | 300 | 0 | 0 | 0 |
| MUC1 | 0 | 300 | 80 | 160 | 240 |
| P21 | 0 | 80 | 0 | 2.5 | 8.3 |
| P27 | 0 | 300 | 5 | 86.7 | 200 |
| P53 | 0 | 100 | 0 | 0 | 0 |
| P-Cadherin | 0 | 300 | 0 | 0 | 20 |
| PR* | 0 | 100 | 0 | 5 | 50 |
| PTEN | 0 | 300 | 0 | 33.3 | 86.7 |
| TACC2 | 0 | 300 | 25 | 50 | 100 |
| TACC3 | 0 | 300 | 50 | 100 | 100 |
| TAU | 0 | 300 | 0 | 0 | 0 |
| TOPO2A | 0 | 65 | 1.7 | 5 | 10 |

*, percent of positive tumor cells
