# Supplementary material for: Protein expression, survival and docetaxel benefit in node-positive breast cancer treated with adjuvant chemotherapy in the FNCLCC - PACS 01 randomized trial
Source: Breast Cancer Res. 2011 Nov 1;13(6):R109. doi: 10.1186/bcr3051 (PMC3326551; doi:10.1186/bcr3051)
Supplement: Additional file 13 — Table S11 (WORD file). Univariate and multivariate analyses "per quartiles" for DFS [file bcr3051-S13.DOC]

**Suppl. Table 11 : Univariate and multivariate analyses “per quartiles” for DFS**

|  | | | | **Univariate** | | | **Multivariate** | | |
| --- | --- | --- | --- | --- | --- | --- | --- | --- | --- |
| **Marker** | **Category** | **Total (N)** | **Event (n)** | **Unadjusted**  **Risk Ratio 95%CI** | **Pr>Chi²** | ***p-*value (log-rank)** | **Adjusted Risk Ratio 95%CI** | **Pr>Chi²** | ***p-*value (Wald)** |
| AF6 | (Min-P25] | 215 | 52 (24%) |  | . | 0.228 |  | . | 0.7 |
|  | (P25-P50] | 304 | 71 (23%) | .956 (0.67,1.37) | 0.8036 |  | 1.04 (0.70,1.54) | 0.8427 |  |
|  | (P50-P75] | 139 | 37 (27%) | 1.15 (0.76,1.76) | 0.5066 |  | .997 (0.62,1.61) | 0.9893 |  |
|  | (P75-Max] | 191 | 34 (18%) | .715 (0.46,1.10) | 0.1285 |  | .806 (0.51,1.28) | 0.3576 |  |
| Angiogenin | (Min-P25] | 240 | 63 (26%) |  | . | 0.889 |  | . | 0.882 |
|  | (P25-P50] | 236 | 57 (24%) | .918 (0.64,1.31) | 0.6414 |  | .857 (0.58,1.27) | 0.4415 |  |
|  | (P50-P75] | 238 | 59 (25%) | .945 (0.66,1.35) | 0.7571 |  | .963 (0.66,1.41) | 0.8468 |  |
|  | (P75-Max] | 236 | 53 (22%) | .865 (0.60,1.25) | 0.4366 |  | .914 (0.62,1.35) | 0.6543 |  |
| Aurora A | (Min-P25] | 585 | 127 (22%) |  | . | 0.051 |  | . | 0.189 |
|  | (P50-P75] | 77 | 22 (29%) | 1.43 (0.91,2.24) | 0.1238 |  | 1.27 (0.77,2.10) | 0.3486 |  |
|  | (P75-Max] | 199 | 58 (29%) | 1.41 (1.03,1.92) | 0.0302 |  | 1.34 (0.96,1.87) | 0.0833 |  |
| BCL2 | (Min-P25] | 377 | 115 (31%) |  | . | <.001 |  | . | 0.165 |
|  | (P25-P50] | 113 | 31 (27%) | .862 (0.58,1.28) | 0.464 |  | 1.14 (0.73,1.76) | 0.5665 |  |
|  | (P50-P75] | 228 | 43 (19%) | .554 (0.39,0.79) | 0.001 |  | .767 (0.51,1.14) | 0.1928 |  |
|  | (P75-Max] | 239 | 43 (18%) | .535 (0.38,0.76) | 0.0005 |  | .702 (0.47,1.06) | 0.0898 |  |
| a-catenin | (Min-P25] | 359 | 92 (26%) |  | . | 0.662 |  | . | 0.47 |
|  | (P25-P50] | 86 | 17 (20%) | .780 (0.47,1.31) | 0.3478 |  | .772 (0.44,1.35) | 0.3663 |  |
|  | (P50-P75] | 244 | 54 (22%) | .849 (0.61,1.19) | 0.3404 |  | .931 (0.65,1.34) | 0.7 |  |
|  | (P75-Max] | 171 | 42 (25%) | .985 (0.68,1.42) | 0.9343 |  | 1.20 (0.81,1.77) | 0.3668 |  |
| b-caten | (Min-P25] | 265 | 72 (27%) |  | . | 0.571 |  | . | 0.889 |
|  | (P25-P50] | 193 | 44 (23%) | .838 (0.58,1.22) | 0.3573 |  | .902 (0.60,1.36) | 0.6216 |  |
|  | (P50-P75] | 222 | 54 (24%) | .895 (0.63,1.27) | 0.5385 |  | .865 (0.58,1.28) | 0.4714 |  |
|  | (P75-Max] | 221 | 46 (21%) | .776 (0.54,1.12) | 0.1789 |  | .883 (0.59,1.32) | 0.542 |  |
| CAV1 | (Min-P25] | 245 | 47 (19%) |  | . | 0.044 |  | . | 0.387 |
|  | (P25-P50] | 245 | 56 (23%) | 1.20 (0.81,1.77) | 0.3571 |  | 1.15 (0.74,1.78) | 0.5433 |  |
|  | (P50-P75] | 228 | 61 (27%) | 1.47 (1.00,2.15) | 0.0487 |  | 1.39 (0.91,2.13) | 0.1312 |  |
|  | (P75-Max] | 239 | 69 (29%) | 1.64 (1.13,2.37) | 0.0091 |  | 1.36 (0.89,2.08) | 0.1606 |  |
| CD10 | (Min-P25] | 409 | 92 (22%) |  | . | 0.48 |  | . | 0.491 |
|  | (P25-P50] | 52 | 16 (31%) | 1.46 (0.86,2.49) | 0.1587 |  | 1.42 (0.81,2.48) | 0.2168 |  |
|  | (P50-P75] | 272 | 68 (25%) | 1.15 (0.84,1.58) | 0.37 |  | 1.24 (0.88,1.73) | 0.217 |  |
|  | (P75-Max] | 184 | 47 (26%) | 1.17 (0.82,1.66) | 0.3809 |  | 1.10 (0.75,1.61) | 0.6323 |  |
| CD44 | (Min-P25] | 430 | 112 (26%) |  | . | 0.494 |  | . | 0.738 |
|  | (P50-P75] | 103 | 22 (21%) | .781 (0.49,1.23) | 0.2888 |  | .827 (0.51,1.35) | 0.4436 |  |
|  | (P75-Max] | 177 | 41 (23%) | .874 (0.61,1.25) | 0.4594 |  | .940 (0.64,1.37) | 0.7506 |  |
| CK14 | (Min-P25] | 774 | 190 (25%) |  | . | 0.974 |  | . | 0.288 |
|  | (P75-Max] | 158 | 39 (25%) | 1.01 (0.71,1.42) | 0.9738 |  | .811 (0.55,1.19) | 0.2879 |  |
| CK5/6 | (Min-P25] | 248 | 66 (27%) |  | . | 0.737 |  | . | 0.614 |
|  | (P25-P50] | 223 | 51 (23%) | .869 (0.60,1.25) | 0.45 |  | .964 (0.65,1.44) | 0.8595 |  |
|  | (P50-P75] | 219 | 50 (23%) | .831 (0.58,1.20) | 0.3229 |  | .864 (0.58,1.30) | 0.4803 |  |
|  | (P75-Max] | 225 | 57 (25%) | .963 (0.68,1.37) | 0.8331 |  | 1.14 (0.77,1.69) | 0.5164 |  |
| CK8/18 | (Min-P25] | 244 | 79 (32%) |  | . | <.001 |  | . | 0.191 |
|  | (P25-P50] | 726 | 160 (22%) | .627 (0.48,0.82) | 0.0007 |  | .815 (0.60,1.11) | 0.1913 |  |
| Cyclin D1 | (Min-P25] | 316 | 83 (26%) |  | . | 0.566 |  | . | 0.514 |
|  | (P25-P50] | 168 | 36 (21%) | .764 (0.52,1.13) | 0.1782 |  | .901 (0.58,1.40) | 0.6435 |  |
|  | (P50-P75] | 241 | 61 (25%) | .943 (0.68,1.31) | 0.7285 |  | 1.23 (0.84,1.79) | 0.2874 |  |
|  | (P75-Max] | 239 | 56 (23%) | .870 (0.62,1.22) | 0.4192 |  | 1.18 (0.79,1.75) | 0.423 |  |
| E-Cadherin | (Min-P25] | 258 | 66 (26%) |  | . | 0.008 |  | . | 0.064 |
|  | (P25-P50] | 253 | 49 (19%) | .740 (0.51,1.07) | 0.11 |  | .710 (0.47,1.06) | 0.0962 |  |
|  | (P50-P75] | 271 | 82 (30%) | 1.24 (0.90,1.72) | 0.1914 |  | 1.13 (0.79,1.62) | 0.5092 |  |
|  | (P75-Max] | 219 | 42 (19%) | .738 (0.50,1.09) | 0.1244 |  | .791 (0.52,1.21) | 0.2819 |  |
| EGFR | (Min-P25] | 814 | 199 (24%) |  | . | 0.895 |  | . | 0.09 |
|  | (P75-Max] | 185 | 45 (24%) | 1.02 (0.74,1.41) | 0.8944 |  | .718 (0.49,1.05) | 0.0895 |  |
| ER | (Min-P25] | 283 | 97 (34%) |  | . | <.001 |  | . | 0.306 |
|  | (P25-P50] | 293 | 70 (24%) | .619 (0.46,0.84) | 0.0022 |  | 1.05 (0.54,2.05) | 0.889 |  |
|  | (P50-P75] | 256 | 50 (20%) | .486 (0.35,0.68) | <.0001 |  | .889 (0.44,1.78) | 0.7409 |  |
|  | (P75-Max] | 236 | 45 (19%) | .482 (0.34,0.69) | <.0001 |  | .701 (0.34,1.43) | 0.3271 |  |
| FGFR1 | (Min-P25] | 185 | 44 (24%) |  | . | 0.733 |  | . | 0.814 |
|  | (P25-P50] | 185 | 46 (25%) | 1.04 (0.69,1.57) | 0.8657 |  | 1.09 (0.70,1.70) | 0.7133 |  |
|  | (P50-P75] | 220 | 48 (22%) | .935 (0.62,1.41) | 0.7477 |  | .944 (0.60,1.48) | 0.8016 |  |
|  | (P75-Max] | 150 | 29 (19%) | .804 (0.50,1.29) | 0.3624 |  | .856 (0.52,1.42) | 0.5448 |  |
| FHIT | (Min-P25] | 237 | 55 (23%) |  | . | 0.01 |  | . | 0.001 |
|  | (P25-P50] | 234 | 67 (29%) | 1.26 (0.88,1.80) | 0.208 |  | 1.59 (1.06,2.40) | 0.0259 |  |
|  | (P50-P75] | 214 | 59 (28%) | 1.21 (0.84,1.74) | 0.3129 |  | 1.83 (1.20,2.77) | 0.0048 |  |
|  | (P75-Max] | 224 | 37 (17%) | .668 (0.44,1.01) | 0.0577 |  | .889 (0.56,1.42) | 0.6237 |  |
| GATA3 | (Min-P25] | 247 | 72 (29%) |  | . | 0.018 |  | . | 0.523 |
|  | (P25-P50] | 248 | 69 (28%) | .933 (0.67,1.30) | 0.6821 |  | .922 (0.63,1.34) | 0.6725 |  |
|  | (P50-P75] | 243 | 47 (19%) | .610 (0.42,0.88) | 0.0085 |  | .721 (0.46,1.13) | 0.153 |  |
|  | (P75-Max] | 244 | 52 (21%) | .679 (0.48,0.97) | 0.0337 |  | .913 (0.59,1.41) | 0.6827 |  |
| Ki67 | (Min-P25] | 238 | 37 (16%) |  | . | <.001 |  | . | 0.069 |
|  | (P25-P50] | 294 | 64 (22%) | 1.45 (0.97,2.17) | 0.0738 |  | 1.30 (0.82,2.06) | 0.2735 |  |
|  | (P50-P75] | 222 | 61 (27%) | 1.94 (1.29,2.93) | 0.0014 |  | 1.67 (1.05,2.65) | 0.0306 |  |
|  | (P75-Max] | 187 | 68 (36%) | 2.80 (1.88,4.18) | <.0001 |  | 1.79 (1.10,2.92) | 0.0189 |  |
| MET | (Min-P25] | 602 | 139 (23%) |  | . | 0.233 |  | . | 0.275 |
|  | (P50-P75] | 98 | 27 (28%) | 1.24 (0.82,1.88) | 0.3013 |  | 1.37 (0.88,2.14) | 0.1624 |  |
|  | (P75-Max] | 217 | 60 (28%) | 1.27 (0.94,1.72) | 0.1211 |  | 1.20 (0.86,1.66) | 0.2795 |  |
| Moesin | (Min-P25] | 824 | 194 (24%) |  | . | 0.145 |  | . | 0.815 |
|  | (P75-Max] | 118 | 34 (29%) | 1.31 (0.91,1.89) | 0.1456 |  | .952 (0.63,1.44) | 0.8148 |  |
| MUC1 | (Min-P25] | 262 | 75 (29%) |  | . | 0.146 |  | . | 0.58 |
|  | (P25-P50] | 269 | 60 (22%) | .750 (0.53,1.05) | 0.0964 |  | .808 (0.55,1.19) | 0.276 |  |
|  | (P50-P75] | 245 | 64 (26%) | .875 (0.63,1.22) | 0.4311 |  | 1.05 (0.72,1.52) | 0.7987 |  |
|  | (P75-Max] | 257 | 53 (21%) | .685 (0.48,0.97) | 0.0353 |  | .979 (0.67,1.43) | 0.9143 |  |
| P21 | (Min-P25] | 378 | 92 (24%) |  | . | 0.008 |  | . | 0.003 |
|  | (P25-P50] | 123 | 37 (30%) | 1.27 (0.87,1.87) | 0.2139 |  | 1.24 (0.82,1.89) | 0.3102 |  |
|  | (P50-P75] | 203 | 31 (15%) | .588 (0.39,0.88) | 0.0105 |  | .450 (0.28,0.73) | 0.0012 |  |
|  | (P75-Max] | 219 | 57 (26%) | 1.09 (0.79,1.52) | 0.5974 |  | 1.01 (0.70,1.44) | 0.975 |  |
| P27 | (Min-P25] | 237 | 66 (28%) |  | . | 0.023 |  | . | 0.612 |
|  | (P25-P50] | 235 | 67 (29%) | 1.03 (0.74,1.45) | 0.8444 |  | 1.20 (0.83,1.73) | 0.3417 |  |
|  | (P50-P75] | 243 | 53 (22%) | .760 (0.53,1.09) | 0.1369 |  | .951 (0.64,1.42) | 0.8047 |  |
|  | (P75-Max] | 228 | 42 (18%) | .614 (0.42,0.90) | 0.0136 |  | .953 (0.62,1.48) | 0.8286 |  |
| P53 | (Min-P25] | 746 | 163 (22%) |  | . | <.001 |  | . | 0.107 |
|  | (P75-Max] | 246 | 79 (32%) | 1.59 (1.21,2.07) | 0.0008 |  | 1.29 (0.95,1.75) | 0.1071 |  |
| P-Cadherin | (Min-P25] | 570 | 125 (22%) |  | . | 0.015 |  | . | 0.234 |
|  | (P50-P75] | 138 | 39 (28%) | 1.33 (0.93,1.91) | 0.1169 |  | 1.32 (0.89,1.97) | 0.1724 |  |
|  | (P75-Max] | 233 | 70 (30%) | 1.51 (1.13,2.03) | 0.0056 |  | 1.30 (0.91,1.86) | 0.1538 |  |
| PR | (Min-P25] | 488 | 153 (31%) |  | . | <.001 |  | . | <.001 |
|  | (P25-P50] | 48 | 17 (35%) | 1.18 (0.72,1.95) | 0.5089 |  | 1.30 (0.73,2.32) | 0.3683 |  |
|  | (P50-P75] | 267 | 56 (21%) | .606 (0.45,0.82) | 0.0013 |  | .765 (0.52,1.13) | 0.1763 |  |
|  | (P75-Max] | 266 | 36 (14%) | .381 (0.26,0.55) | <.0001 |  | .449 (0.29,0.70) | 0.0004 |  |
| PTEN | (Min-P25] | 314 | 75 (24%) |  | . | 0.738 |  | . | 0.397 |
|  | (P25-P50] | 177 | 36 (20%) | .858 (0.58,1.28) | 0.4511 |  | .788 (0.52,1.20) | 0.2681 |  |
|  | (P50-P75] | 206 | 52 (25%) | 1.06 (0.74,1.50) | 0.7625 |  | 1.06 (0.71,1.58) | 0.7663 |  |
|  | (P75-Max] | 227 | 57 (25%) | 1.07 (0.76,1.51) | 0.6958 |  | 1.14 (0.79,1.65) | 0.4727 |  |
| TACC2 | (Min-P25] | 269 | 60 (22%) |  | . | 0.411 |  | . | 0.781 |
|  | (P25-P50] | 171 | 46 (27%) | 1.23 (0.84,1.81) | 0.2921 |  | 1.23 (0.80,1.88) | 0.343 |  |
|  | (P50-P75] | 251 | 67 (27%) | 1.26 (0.89,1.78) | 0.1939 |  | 1.17 (0.79,1.72) | 0.4371 |  |
|  | (P75-Max] | 180 | 38 (21%) | .972 (0.65,1.46) | 0.8894 |  | 1.07 (0.69,1.67) | 0.7556 |  |
| TACC3 | (Min-P25] | 159 | 34 (21%) |  | . | 0.792 |  | . | 0.998 |
|  | (P25-P50] | 252 | 64 (25%) | 1.20 (0.79,1.81) | 0.4007 |  | .995 (0.63,1.56) | 0.9811 |  |
|  | (P50-P75] | 185 | 39 (21%) | 1.02 (0.64,1.61) | 0.9423 |  | 1.02 (0.62,1.66) | 0.9463 |  |
|  | (P75-Max] | 3 | 1 (33%) | 1.38 (0.19,10.1) | 0.7526 |  | 1.21 (0.16,8.96) | 0.854 |  |
| TAU | (Min-P25] | 685 | 171 (25%) |  | . | 0.011 |  | . | 0.243 |
|  | (P75-Max] | 141 | 22 (16%) | .567 (0.36,0.88) | 0.0124 |  | .754 (0.47,1.21) | 0.2428 |  |
| TOPO2A | (Min-P25] | 230 | 42 (18%) |  | . | <.001 |  | . | 0.001 |
|  | (P25-P50] | 326 | 81 (25%) | 1.41 (0.97,2.04) | 0.0725 |  | 1.47 (0.97,2.23) | 0.0707 |  |
|  | (P50-P75] | 193 | 39 (20%) | 1.14 (0.74,1.76) | 0.563 |  | 1.00 (0.62,1.64) | 0.9877 |  |
|  | (P75-Max] | 164 | 59 (36%) | 2.33 (1.57,3.46) | <.0001 |  | 2.13 (1.36,3.33) | 0.0009 |  |
