# Supplementary material for: Protein expression, survival and docetaxel benefit in node-positive breast cancer treated with adjuvant chemotherapy in the FNCLCC - PACS 01 randomized trial
Source: Breast Cancer Res. 2011 Nov 1;13(6):R109. doi: 10.1186/bcr3051 (PMC3326551; doi:10.1186/bcr3051)
Supplement: Additional file 14 — Table S12 (WORD file). Univariate and multivariate analyses "per quartiles" for interaction with chemotherapy arm. [file bcr3051-S14.DOC]

**Suppl. Table 12 : Univariate and multivariate analyses “per quartiles”for interaction with chemotherapy arm.**

|  |  |  |  | **Univariate** | | **Multivariate** | |
| --- | --- | --- | --- | --- | --- | --- | --- |
| **Marker** | **Category** | **Arm** | **Total (N)** | **Unadjusted Risk Ratio 95%CI** | ***p-*value for Interaction** | **Adjusted Risk Ratio 95%CI** | ***p-*value for Interaction** |
| AF6 | (Min-P25] | FEC | 113 |  | 0.59 |  | 0.624 |
|  |  | FEC-D | 102 | .952 (0.55,1.64) |  | .936 (0.51,1.73) |  |
|  | (P25-P50] | FEC | 133 |  |  |  |  |
|  |  | FEC-D | 171 | .848 (0.53,1.35) |  | 1.13 (0.68,1.88) |  |
|  | (P50-P75] | FEC | 82 |  |  |  |  |
|  |  | FEC-D | 57 | .981 (0.51,1.89) |  | .955 (0.45,2.03) |  |
|  | (P75-Max] | FEC | 92 |  |  |  |  |
|  |  | FEC-D | 99 | .545 (0.27,1.09) |  | .574 (0.28,1.20) |  |
| Angiogenin | (Min-P25] | FEC | 122 |  | 0.982 |  | 0.797 |
|  |  | FEC-D | 118 | .692 (0.42,1.15) |  | .758 (0.43,1.32) |  |
|  | (P25-P50] | FEC | 109 |  |  |  |  |
|  |  | FEC-D | 127 | .664 (0.39,1.12) |  | .779 (0.43,1.40) |  |
|  | (P50-P75] | FEC | 121 |  |  |  |  |
|  |  | FEC-D | 117 | .736 (0.44,1.23) |  | .668 (0.38,1.19) |  |
|  | (P75-Max] | FEC | 121 |  |  |  |  |
|  |  | FEC-D | 115 | .631 (0.36,1.09) |  | .534 (0.29,0.97) |  |
| Aurora A | (Min-P25] | FEC | 293 |  | 0.197 |  | 0.305 |
|  |  | FEC-D | 292 | .874 (0.62,1.24) |  | .907 (0.62,1.33) |  |
|  | (P50-P75] | FEC | 34 |  |  |  |  |
|  |  | FEC-D | 43 | .477 (0.20,1.12) |  | .652 (0.24,1.76) |  |
|  | (P75-Max] | FEC | 101 |  |  |  |  |
|  |  | FEC-D | 98 | .536 (0.31,0.92) |  | .514 (0.29,0.92) |  |
| **α**-Catenin | (Min-P25] | FEC | 177 |  | 0.442 |  | 0.186 |
|  |  | FEC-D | 182 | .771 (0.51,1.16) |  | .853 (0.53,1.37) |  |
|  | (P25-P50] | FEC | 45 |  |  |  |  |
|  |  | FEC-D | 41 | 1.20 (0.46,3.10) |  | 1.02 (0.33,3.17) |  |
|  | (P50-P75] | FEC | 111 |  |  |  |  |
|  |  | FEC-D | 133 | .877 (0.51,1.50) |  | 1.06 (0.60,1.88) |  |
|  | (P75-Max] | FEC | 94 |  |  |  |  |
|  |  | FEC-D | 77 | .502 (0.26,0.97) |  | .450 (0.22,0.92) |  |
| **β**-Catenin | (Min-P25] | FEC | 123 |  | 0.392 |  | 0.21 |
|  |  | FEC-D | 142 | .941 (0.59,1.49) |  | 1.19 (0.69,2.05) |  |
|  | (P25-P50] | FEC | 93 |  |  |  |  |
|  |  | FEC-D | 100 | .544 (0.30,1.00) |  | .597 (0.31,1.16) |  |
|  | (P50-P75] | FEC | 120 |  |  |  |  |
|  |  | FEC-D | 102 | .847 (0.49,1.45) |  | 1.02 (0.56,1.84) |  |
|  | (P75-Max] | FEC | 111 |  |  |  |  |
|  |  | FEC-D | 110 | .568 (0.31,1.03) |  | .497 (0.26,0.94) |  |
| BCL2 | (Min-P25] | FEC | 184 |  | 0.895 |  | 0.601 |
|  |  | FEC-D | 193 | .716 (0.50,1.03) |  | .778 (0.52,1.17) |  |
|  | (P25-P50] | FEC | 56 |  |  |  |  |
|  |  | FEC-D | 57 | .826 (0.40,1.69) |  | 1.06 (0.48,2.37) |  |
|  | (P50-P75] | FEC | 112 |  |  |  |  |
|  |  | FEC-D | 116 | .586 (0.32,1.08) |  | .553 (0.28,1.08) |  |
|  | (P75-Max] | FEC | 117 |  |  |  |  |
|  |  | FEC-D | 122 | .763 (0.42,1.40) |  | 1.04 (0.54,2.01) |  |
| CAV1 | (Min-P25] | FEC | 113 |  | 0.672 |  | 0.622 |
|  |  | FEC-D | 132 | .964 (0.54,1.71) |  | 1.05 (0.54,2.07) |  |
|  | (P25-P50] | FEC | 124 |  |  |  |  |
|  |  | FEC-D | 121 | .614 (0.36,1.05) |  | .627 (0.35,1.13) |  |
|  | (P50-P75] | FEC | 121 |  |  |  |  |
|  |  | FEC-D | 107 | .638 (0.38,1.07) |  | .744 (0.44,1.27) |  |
|  | (P75-Max] | FEC | 113 |  |  |  |  |
|  |  | FEC-D | 126 | .729 (0.45,1.17) |  | .684 (0.41,1.15) |  |
| CD10 | (Min-P25] | FEC | 197 |  | 0.674 |  | 0.819 |
|  |  | FEC-D | 212 | .811 (0.54,1.22) |  | .874 (0.55,1.38) |  |
|  | (P25-P50] | FEC | 26 |  |  |  |  |
|  |  | FEC-D | 26 | .525 (0.19,1.45) |  | .630 (0.20,1.97) |  |
|  | (P50-P75] | FEC | 145 |  |  |  |  |
|  |  | FEC-D | 127 | .630 (0.38,1.03) |  | .752 (0.44,1.28) |  |
|  | (P75-Max] | FEC | 88 |  |  |  |  |
|  |  | FEC-D | 96 | .912 (0.51,1.63) |  | .911 (0.48,1.74) |  |
| CD44 | (Min-P25] | FEC | 210 |  | 0.117 |  | 0.185 |
|  |  | FEC-D | 220 | .682 (0.47,0.99) |  | .631 (0.41,0.96) |  |
|  | (P50-P75] | FEC | 48 |  |  |  |  |
|  |  | FEC-D | 55 | 1.87 (0.76,4.59) |  | 1.88 (0.71,4.99) |  |
|  | (P75-Max] | FEC | 80 |  |  |  |  |
|  |  | FEC-D | 97 | .683 (0.37,1.26) |  | .831 (0.43,1.59) |  |
| CK14 | (Min-P25] | FEC | 386 |  | 0.506 |  | 0.142 |
|  |  | FEC-D | 388 | .704 (0.53,0.94) |  | .769 (0.56,1.05) |  |
|  | (P75-Max] | FEC | 84 |  |  |  |  |
|  |  | FEC-D | 74 | .551 (0.28,1.07) |  | .406 (0.18,0.90) |  |
| CK5/6 | (Min-P25] | FEC | 122 |  | 0.928 |  | 0.594 |
|  |  | FEC-D | 126 | .763 (0.47,1.24) |  | .844 (0.48,1.48) |  |
|  | (P25-P50] | FEC | 105 |  |  |  |  |
|  |  | FEC-D | 118 | .839 (0.48,1.45) |  | .997 (0.56,1.78) |  |
|  | (P50-P75] | FEC | 121 |  |  |  |  |
|  |  | FEC-D | 98 | .733 (0.41,1.30) |  | .742 (0.39,1.42) |  |
|  | (P75-Max] | FEC | 106 |  |  |  |  |
|  |  | FEC-D | 119 | .649 (0.38,1.10) |  | .533 (0.30,0.94) |  |
| CK8/18 | (Min-P25] | FEC | 123 |  | 0.283 |  | 0.746 |
|  |  | FEC-D | 121 | .835 (0.54,1.30) |  | .772 (0.48,1.25) |  |
|  | (P25-P50] | FEC | 358 |  |  |  |  |
|  |  | FEC-D | 368 | .620 (0.45,0.85) |  | .690 (0.49,0.97) |  |
| MET | (Min-P25] | FEC | 301 |  | 0.202 |  | 0.255 |
|  |  | FEC-D | 301 | .852 (0.61,1.19) |  | .903 (0.63,1.30) |  |
|  | (P50-P75] | FEC | 45 |  |  |  |  |
|  |  | FEC-D | 53 | .805 (0.37,1.74) |  | .898 (0.38,2.15) |  |
|  | (P75-Max] | FEC | 105 |  |  |  |  |
|  |  | FEC-D | 112 | .486 (0.29,0.82) |  | .521 (0.30,0.91) |  |
| Cyclin D1 | (Min-P25] | FEC | 161 |  | 0.735 |  | 0.473 |
|  |  | FEC-D | 155 | .715 (0.46,1.10) |  | .777 (0.48,1.26) |  |
|  | (P25-P50] | FEC | 86 |  |  |  |  |
|  |  | FEC-D | 82 | .745 (0.39,1.44) |  | 1.10 (0.52,2.33) |  |
|  | (P50-P75] | FEC | 117 |  |  |  |  |
|  |  | FEC-D | 124 | .947 (0.57,1.56) |  | .962 (0.56,1.65) |  |
|  | (P75-Max] | FEC | 114 |  |  |  |  |
|  |  | FEC-D | 125 | .631 (0.37,1.08) |  | .502 (0.28,0.91) |  |
| E-Cadherin | (Min-P25] | FEC | 131 |  | 0.727 |  | 0.89 |
|  |  | FEC-D | 127 | .744 (0.46,1.21) |  | .775 (0.44,1.37) |  |
|  | (P25-P50] | FEC | 121 |  |  |  |  |
|  |  | FEC-D | 132 | .558 (0.31,0.99) |  | .690 (0.38,1.26) |  |
|  | (P50-P75] | FEC | 135 |  |  |  |  |
|  |  | FEC-D | 136 | .846 (0.55,1.31) |  | .837 (0.53,1.33) |  |
|  | (P75-Max] | FEC | 107 |  |  |  |  |
|  |  | FEC-D | 112 | .690 (0.37,1.27) |  | .588 (0.30,1.15) |  |
| EGFR | (Min-P25] | FEC | 401 |  | 0.154 |  | 0.235 |
|  |  | FEC-D | 413 | .841 (0.64,1.11) |  | .851 (0.63,1.16) |  |
|  | (P75-Max] | FEC | 95 |  |  |  |  |
|  |  | FEC-D | 90 | .516 (0.28,0.95) |  | .582 (0.30,1.13) |  |
| FGFR1 | (Min-P25] | FEC | 89 |  | 0.599 |  | 0.957 |
|  |  | FEC-D | 96 | .654 (0.36,1.19) |  | .761 (0.40,1.46) |  |
|  | (P25-P50] | FEC | 98 |  |  |  |  |
|  |  | FEC-D | 87 | .922 (0.52,1.65) |  | .889 (0.46,1.72) |  |
|  | (P50-P75] | FEC | 105 |  |  |  |  |
|  |  | FEC-D | 115 | .814 (0.46,1.43) |  | .650 (0.35,1.22) |  |
|  | (P75-Max] | FEC | 70 |  |  |  |  |
|  |  | FEC-D | 80 | .500 (0.24,1.06) |  | .860 (0.39,1.90) |  |
| FHIT | (Min-P25] | FEC | 127 |  | 0.068 |  | 0.329 |
|  |  | FEC-D | 110 | 1.18 (0.70,2.00) |  | .997 (0.52,1.90) |  |
|  | (P25-P50] | FEC | 109 |  |  |  |  |
|  |  | FEC-D | 125 | .912 (0.56,1.47) |  | .974 (0.58,1.65) |  |
|  | (P50-P75] | FEC | 101 |  |  |  |  |
|  |  | FEC-D | 113 | .459 (0.27,0.78) |  | .514 (0.29,0.90) |  |
|  | (P75-Max] | FEC | 111 |  |  |  |  |
|  |  | FEC-D | 113 | .612 (0.31,1.20) |  | .731 (0.36,1.49) |  |
| GATA3 | (Min-P25] | FEC | 116 |  | 0.985 |  | 0.815 |
|  |  | FEC-D | 131 | .731 (0.46,1.16) |  | .778 (0.47,1.29) |  |
|  | (P25-P50] | FEC | 133 |  |  |  |  |
|  |  | FEC-D | 115 | .767 (0.48,1.24) |  | .878 (0.51,1.50) |  |
|  | (P50-P75] | FEC | 125 |  |  |  |  |
|  |  | FEC-D | 118 | .683 (0.38,1.22) |  | .716 (0.37,1.37) |  |
|  | (P75-Max] | FEC | 121 |  |  |  |  |
|  |  | FEC-D | 123 | .676 (0.39,1.18) |  | .655 (0.35,1.24) |  |
| Ki67 | (Min-P25] | FEC | 116 |  | 0.901 |  | 0.401 |
|  |  | FEC-D | 122 | .758 (0.40,1.45) |  | 1.24 (0.59,2.63) |  |
|  | (P25-P50] | FEC | 142 |  |  |  |  |
|  |  | FEC-D | 152 | .807 (0.49,1.32) |  | .970 (0.56,1.67) |  |
|  | (P50-P75] | FEC | 121 |  |  |  |  |
|  |  | FEC-D | 101 | .638 (0.38,1.08) |  | .684 (0.39,1.20) |  |
|  | (P75-Max] | FEC | 95 |  |  |  |  |
|  |  | FEC-D | 92 | .652 (0.40,1.06) |  | .573 (0.34,0.97) |  |
| Moesin | (Min-P25] | FEC | 412 |  | 0.849 |  | 0.92 |
|  |  | FEC-D | 412 | .722 (0.54,0.96) |  | .774 (0.57,1.05) |  |
|  | (P75-Max] | FEC | 56 |  |  |  |  |
|  |  | FEC-D | 62 | .775 (0.40,1.52) |  | .798 (0.39,1.65) |  |
| MUC1 | (Min-P25] | FEC | 135 |  | 0.14 |  | 0.479 |
|  |  | FEC-D | 127 | 1.07 (0.68,1.68) |  | 1.09 (0.66,1.82) |  |
|  | (P25-P50] | FEC | 137 |  |  |  |  |
|  |  | FEC-D | 132 | .706 (0.42,1.18) |  | .657 (0.37,1.17) |  |
|  | (P50-P75] | FEC | 115 |  |  |  |  |
|  |  | FEC-D | 130 | .486 (0.29,0.80) |  | .562 (0.32,0.99) |  |
|  | (P75-Max] | FEC | 127 |  |  |  |  |
|  |  | FEC-D | 130 | .637 (0.37,1.10) |  | .693 (0.39,1.23) |  |
| P21 | (Min-P25] | FEC | 187 |  | 0.023 |  | 0.052 |
|  |  | FEC-D | 191 | .746 (0.49,1.13) |  | .738 (0.48,1.14) |  |
|  | (P25-P50] | FEC | 53 |  |  |  |  |
|  |  | FEC-D | 70 | .340 (0.17,0.66) |  | .402 (0.19,0.86) |  |
|  | (P50-P75] | FEC | 106 |  |  |  |  |
|  |  | FEC-D | 97 | 1.58 (0.77,3.22) |  | 1.84 (0.74,4.55) |  |
|  | (P75-Max] | FEC | 113 |  |  |  |  |
|  |  | FEC-D | 106 | .674 (0.40,1.14) |  | .639 (0.35,1.16) |  |
| P27 | (Min-P25] | FEC | 112 |  | 0.444 |  | 0.462 |
|  |  | FEC-D | 125 | .571 (0.35,0.93) |  | .658 (0.38,1.14) |  |
|  | (P25-P50] | FEC | 118 |  |  |  |  |
|  |  | FEC-D | 117 | .844 (0.52,1.36) |  | .860 (0.52,1.43) |  |
|  | (P50-P75] | FEC | 121 |  |  |  |  |
|  |  | FEC-D | 122 | .951 (0.55,1.63) |  | 1.08 (0.60,1.95) |  |
|  | (P75-Max] | FEC | 105 |  |  |  |  |
|  |  | FEC-D | 123 | .593 (0.32,1.10) |  | .485 (0.25,0.93) |  |
| P53 | (Min-P25] | FEC | 364 |  | 0.974 |  | 0.74 |
|  |  | FEC-D | 382 | .708 (0.52,0.96) |  | .763 (0.54,1.08) |  |
|  | (P75-Max] | FEC | 123 |  |  |  |  |
|  |  | FEC-D | 123 | .701 (0.45,1.10) |  | .710 (0.45,1.13) |  |
| P-Cadherin | (Min-P25] | FEC | 282 |  | 0.5 |  | 0.869 |
|  |  | FEC-D | 288 | .823 (0.58,1.17) |  | .785 (0.52,1.17) |  |
|  | (P50-P75] | FEC | 66 |  |  |  |  |
|  |  | FEC-D | 72 | .776 (0.41,1.46) |  | .497 (0.24,1.04) |  |
|  | (P75-Max] | FEC | 113 |  |  |  |  |
|  |  | FEC-D | 120 | .579 (0.36,0.93) |  | .703 (0.43,1.14) |  |
| PTEN | (Min-P25] | FEC | 152 |  | 0.093 |  | 0.348 |
|  |  | FEC-D | 162 | 1.04 (0.66,1.64) |  | .995 (0.60,1.64) |  |
|  | (P25-P50] | FEC | 87 |  |  |  |  |
|  |  | FEC-D | 90 | .371 (0.18,0.75) |  | .432 (0.20,0.91) |  |
|  | (P50-P75] | FEC | 110 |  |  |  |  |
|  |  | FEC-D | 96 | .903 (0.52,1.56) |  | .877 (0.45,1.70) |  |
|  | (P75-Max] | FEC | 118 |  |  |  |  |
|  |  | FEC-D | 109 | .656 (0.38,1.12) |  | .702 (0.40,1.24) |  |
| ER | (Min-P25] | FEC | 137 |  | 0.846 |  | 0.998 |
|  |  | FEC-D | 146 | .630 (0.42,0.94) |  | .794 (0.52,1.22) |  |
|  | (P25-P50] | FEC | 159 |  |  |  |  |
|  |  | FEC-D | 134 | .831 (0.52,1.34) |  | .754 (0.45,1.25) |  |
|  | (P50-P75] | FEC | 116 |  |  |  |  |
|  |  | FEC-D | 140 | .760 (0.44,1.32) |  | .784 (0.43,1.43) |  |
|  | (P75-Max] | FEC | 119 |  |  |  |  |
|  |  | FEC-D | 117 | .734 (0.41,1.33) |  | .827 (0.40,1.73) |  |
| PR | (Min-P25] | FEC | 255 |  | 0.56 |  | 0.843 |
|  |  | FEC-D | 233 | .853 (0.62,1.17) |  | .863 (0.61,1.22) |  |
|  | (P25-P50] | FEC | 27 |  |  |  |  |
|  |  | FEC-D | 21 | .544 (0.20,1.48) |  | .453 (0.14,1.47) |  |
|  | (P50-P75] | FEC | 118 |  |  |  |  |
|  |  | FEC-D | 149 | .721 (0.43,1.22) |  | .809 (0.46,1.44) |  |
|  | (P75-Max] | FEC | 131 |  |  |  |  |
|  |  | FEC-D | 135 | .528 (0.27,1.04) |  | .685 (0.32,1.46) |  |
| TACC2 | (Min-P25] | FEC | 132 |  | 0.064 |  | 0.037 |
|  |  | FEC-D | 137 | .774 (0.47,1.29) |  | .695 (0.39,1.24) |  |
|  | (P25-P50] | FEC | 98 |  |  |  |  |
|  |  | FEC-D | 73 | .803 (0.44,1.45) |  | .854 (0.45,1.62) |  |
|  | (P50-P75] | FEC | 125 |  |  |  |  |
|  |  | FEC-D | 126 | 1.20 (0.74,1.95) |  | 1.55 (0.91,2.63) |  |
|  | (P75-Max] | FEC | 81 |  |  |  |  |
|  |  | FEC-D | 99 | .388 (0.20,0.76) |  | .430 (0.21,0.88) |  |
| TACC3 | (Min-P25] | FEC | 70 |  | 0.166 |  | 0.195 |
|  |  | FEC-D | 89 | 1.22 (0.61,2.43) |  | 1.15 (0.56,2.35) |  |
|  | (P25-P50] | FEC | 135 |  |  |  |  |
|  |  | FEC-D | 117 | .986 (0.60,1.61) |  | 1.20 (0.69,2.09) |  |
|  | (P50-Max] | FEC | 92 |  |  |  |  |
|  |  | FEC-D | 96 | .550 (0.27,0.99) |  | 0.58 (0.28,1.11) |  |
| TAU | (Min-P25] | FEC | 340 |  | 0.536 |  | 0.611 |
|  |  | FEC-D | 345 | .746 (0.55,1.01) |  | .828 (0.59,1.16) |  |
|  | (P75-Max] | FEC | 65 |  |  |  |  |
|  |  | FEC-D | 76 | .989 (0.43,2.29) |  | .964 (0.38,2.42) |  |
| TOPO2A | (Min-P25] | FEC | 119 |  | 0.626 |  | 0.976 |
|  |  | FEC-D | 111 | 1.06 (0.58,1.94) |  | .803 (0.40,1.62) |  |
|  | (P25-P50] | FEC | 171 |  |  |  |  |
|  |  | FEC-D | 155 | .677 (0.43,1.06) |  | .748 (0.46,1.22) |  |
|  | (P50-P75] | FEC | 98 |  |  |  |  |
|  |  | FEC-D | 95 | .677 (0.36,1.28) |  | .801 (0.40,1.62) |  |
|  | (P75-Max] | FEC | 75 |  |  |  |  |
|  |  | FEC-D | 89 | .662 (0.40,1.10) |  | .716 (0.41,1.24) |  |
